# Behavioral and molecular effects of micro and nanoplastics across three plastic types in fish: weathered microfibers induce a similar response to nanosized particles

**DOI:** 10.3389/ftox.2024.1490223

**Published:** 2024-11-26

**Authors:** Sara J. Hutton, Lauren Kashiwabara, Erin Anderson, Samreen Siddiqui, Bryan Harper, Stacey Harper, Susanne M. Brander

**Affiliations:** ^1^ Department of Environmental and Molecular Toxicology, Oregon State University, Corvallis, OR, United States; ^2^ Fisheries, Wildlife, and Conservation Sciences Department; Coastal Oregon Marine Experiment Station, Hatfield Marine Science Center, Oregon State University, Newport, OR, United States; ^3^ School of Chemical, Biological and Environmental Engineering, Oregon State University, Corvallis, OR, United States

**Keywords:** microplastic, Inland Silverside, behavioral toxicology, RNA seq, nanoplastic, microfiber, gene ontology

## Abstract

Micro and nanoplastics (MNPs) are ubiquitous in the environment and have been detected in most ecosystems, including remote regions. The class of contaminants under the MNP umbrella is quite broad and encompasses variable polymer types, shapes, and sizes. Fibers are the most frequently detected in the environment, followed by fragments, but still represent only a small fraction of laboratory studies. Many toxicity studies have been done using polystyrene microbeads which represent neither the polymer nor shape most present in the environment. Additionally, most of these studies are done using virgin particles when the majority of MNP pollution is from secondary microplastics which have weathered and broken down over time. To address these data gaps, we exposed the model fish Inland Silverside, *Menidia beryllina*, for 21-days to micro and nano cryo-milled tire particles, micro and nano polylactic acid, and polyester microfibers, both weathered and unweathered treatments were tested. We evaluated the impacts of these particles on growth, behavior, and gene expression to compare the relative toxicities of the different particles. We found that overall, the nanoparticles and weathered fibers had the greatest effect on behavior and gene expression. Gene ontology analysis revealed strong evidence suggesting MNP exposure affected pathways involved in muscle contraction and function. Unweathered microfibers decreased growth which may be a result of food dilution. Our results also suggest that under weathering conditions polyester microfibers breakdown into smaller sizes and induce toxicity similar to nanoparticles. This study highlights the variable effects of MNPs in fish and emphasizes the importance of considering particle shape and size in toxicity studies.

## 1 Introduction

Despite the rapid increase in toxicity studies of micro (1 µm–5 mm) and nanoplastics (<1000 nm), there remains a large knowledge gap regarding differential toxicity between weathered and unweathered plastics. Plastic becomes weathered and broken down through hydrolytic, thermo-oxidative, microbial, enzymatic, chemical, and UV-photodegradation. MNPs enter the aquatic environment through human activity and improper waste disposal or are formed from larger macroplastics that breakdown through weathering. As MNPs travel through aquatic ecosystems, they are exposed to UV-A and UV-B radiation from sunlight. Furthermore, if MNPs pass through wastewater treatment plants (Sun et al., 2019) they may undergo exposure to UV-C radiation, which is used as a method for disinfection (Cerreta et al., 2020). UV radiation alters the chemical composition of plastics and their leachates (Simon et al., 2021). For instance, metabolism of chemicals from additives, plasticizers, stabilizers, and other chemicals may alter leachate toxicity. In the aquatic environment, MNPs are also exposed to mechanical processes, such as wave action and biofilm formation, which can increase degradation and weathering (Cooper and Corcoran, 2010; Rummel et al., 2017; Zbyszewski et al., 2014).

This study examined the effects of three MNPs found in aquatic environments: polylactic acid (PLA), cryo-milled tire particles (TP), and polyester microfibers (MF). Fibers made up 80% of the particle collected in a study of the San Francisco Bay, followed by fragments at 17% (Sutton et al., 2016). Fibers are also commonly found in biota (Caldwell et al., 2022; Lasdin et al., 2023; Torres et al., 2023). Tire particles (TPs) have also been identified as a prevalent microplastic in estuarine samples and have been identified as a major contribution to MNP pollution (Gray et al., 2018; Werbowski et al., 2021). PLA is from plant-based polymers and is biodegradable under industrial composting conditions, however it does not fully degrade in aquatic environments but can breakdown faster than petroleum-based polymers (Ali et al., 2023). Nanoplastics are more challenging to quantify in the environment than microplastics, as technologies are not readily available to reliably measure nanoparticles in field samples. A recent study found that of the 2.4 ± 1.3 × 10^5^ plastic particles/liter found in bottled water, approximately 90% were nanoplastics (Qian et al., 2024). While a variety of methods have been published demonstrating the ability to measure nanoparticles in environmental samples (Moon et al., 2024), their fate and transport remains a large knowledge gap.

Here, we assessed the impacts of various micro and nanoplastics (MNPs) on fish, emphasizing the importance of considering particle shape and size in toxicity studies. TPs are frequently detected within the gut of estuarine fish species (Parker et al., 2020) and laboratory toxicity testing suggests they can elicit sublethal effects such as changes in behavior and oxidative stress (Brander et al., 2024a; Chibwe et al., 2022; Shengchen et al., 2021; Siddiqui et al., 2022; 2023). Some species of fish appear to be especially sensitive to weathering effects of TPs. For example, Coho salmon (*Oncorhynchus kisutch*) have been found to be particularly sensitive to a metabolite formed from a tire rubber antioxidant under UV exposure which is found in streams due to road runoff of tires (Tian et al., 2021). Zebrafish (*Danio rerio*) larvae exposed to PLA showed increased anxiety-like behavior and inhibited acetylcholinesterase activity (de Oliveira et al., 2021). Degraded PLA has also been found to induce oxidative stress, apoptosis, and impair mitochondria in developing Zebrafish (Zhang et al., 2021). Microfibers are one of the most commonly detected types of anthropogenic particles (Boisen et al., 2024; Granek et al., 2022) and also appear to elicit greater toxicity (e.g., food dilution, respiratory stress) than other microplastic types (Brander et al., 2024a; Siddiqui et al., 2022; 2023; Stienbarger et al., 2021). These findings underscore the complex nature of MNP toxicity and highlight the need for further research to fully understand their environmental and biological impacts.

The vast majority of laboratory toxicity studies are still conducted using commercially available polystyrene microspheres, but relatively few studies are done with more environmentally relevant polymers. Given the overall scarcity of comparative data on other polymers’ potential effects in fish, especially PLA and synthetic microfibers, we exposed the model estuarine species, Inland Silverside (*Menidia beryllina*), to micro and nano TPs and PLA, and polyester microfibers for 21-days. In addition, all plastic types had an unweathered and a weathered treatment. Inland Silversides are a U.S. EPA approved model estuarine fish used for regulatory and research testing with demonstrated sensitivity to MNP exposure in early life (Athey et al., 2020; Brander et al., 2012; Siddiqui et al., 2022; Siddiqui et al., 2023). We were specifically interested in studying an estuarine species since estuaries act as sinks for many contaminants, including MNPs, and are critical breeding grounds, nurseries, and habitat for many fishes (Méjanelle et al., 2020). We explored the effects of these MNPs on larval growth, behavior, and gene expression. We sought to focus our chronic exposures on the larval stage of the fish’s life history, as early development is often the most sensitive life stage.

## 2 Methods

### 2.1 Particle generation

We exposed *M. beryllina* to cryomilled TPs, PLA, and polyester MFs made in house per protocols described in Cunningham et al. (2022), McColley et al. (2023), and Siddiqui et al. (2023), respectively. The TP and PLA exposures had both a micro and nano size fraction and the MF exposures had only a micro size fraction. In addition, each treatment had an unweathered and a weathered counterpart. To create weathered particles that test materials were irradiated for 3 days by UV-A/B at 280–400 nm and UV-C at 250 nm on a shaker table, suspended in 15 PSU seawater to achieve weathered conditions.

#### 2.1.1 Particle identification/confirmation

All scanning electron microscopy (SEM) and associated prep were done using the Oregon State University (OSU) Electron Microscopy Facility (EMF). Samples were mounted on to a specimen stub and secured with carbon tape. They were sputter coated with a thin Au/Pd layer (Cressington108 auto/SE sample coater) to improve conductivity and SEM quality. Polyester microfibers were imaged using a FEI Quanta 3D dual beam SEM/FIB at 5.00 kV. Tire particles and PLA particles were imaged using Helios 650 Ultra Resolution Dual Beam FEG SEM 5.00 kV.

All particle types, PLA, TPs, and polyester MFs, were analyzed via Fourier transform infrared spectroscopy (FTIR) using Attenuated Total Reflectance (ATR), as described in Lasdin et al. (2023). Briefly, PLA was analyzed using a diamond tip and 16 scans due to its powdered form. TPs and polyester MFs were confirmed using μFTIR at 128 scans with a germanium tip. All particle types were confirmed (>80%). All spectra were corrected using atmospheric suppression and background scans were taken for each run (Primpke et al., 2018).

### 2.2 Organism husbandry

Adult Inland Silverside broodstock were housed and spawned at the (OSU), Hatfield Marine Science Center and transferred to Corvallis, OR where the experimental exposures were conducted under Animal Care and Use Program (ACUP) protocol #4999. Adult Inland Silverside broodstock were maintained at 10–20 PSU and 23°C on a 14:10 light cycle. Adult fish were fed a combination of Hikari tropical micro pellets (Kyorin Food Industries Ltd., Kasai City, Japan), Hikari freeze-dried tubifex worms (Kyorin Food Industries Ltd), Hikari frozen mysid shrimp (Kyorin Food Industries Ltd., Kasai City, Japan), and live Artemia nauplii hatched from Brine Shrimp Eggs (Brine Shrimp Direct, Ogden, UT, United States) supplemented with Selcon™ (American Marine Inc., Ridgefield, CT, United States). Adult Inland Silversides were approximately 1.5–2 years old at the time of spawning. The spawning protocol was adapted from (Middaugh et al., 1986; DeCourten and Brander, 2017) and occurred as described in (Hutton et al., 2021). Briefly, substrate was added to adult broodstock tanks for 16–20 h following which time it was removed and placed into glass jars in a cooler to maintain the temperature of the water. Spawning substrate and embryos were subsequently transported to OSU, Corvallis, OR main campus, placed into 15 PSU artificial seawater (ASW) created with Instant Ocean and reverse osmosis water, and allowed to develop on the substrate until 5 days post fertilization (dpf). At 5 dpf, embryos were gently removed from the spawning substrate using forceps, placed into ASW at 15 PSU, rinsed, and assessed for development using a VWR VistaVision Dissecting Scope (VWR International, Radnor, PA, United States).

### 2.3 Experimental exposure

Microplastic contamination was prevented by following recommendations from Brander et al. (2020) and Cowger et al. (2020). Specifically, orange cotton lab coats were worn at all times, all beakers were covered with parafilm, and a HEPA air-filter was run throughout the exposures. Additionally, control filters were left open and exposed to the air to verify background contamination (Siddiqui et al., 2023). Inland Silverside embryos, 5 dpf, were placed into 500 mL of exposure solution. Embryos hatched at approximately 7-8 dpf, 2-3 days into the exposure. There were 8–10 organisms per replicate (n = 3). The exposure solutions were semi-static with 50% water renewal every 48 h and were maintained on constant aeration. During each water change debris were removed and pH, temperature, salinity, dissolved oxygen, and ammonia were measured from new and old exposure solutions. Water quality throughout the exposure was as follows (mean ± standard error): pH (8.10 ± 0.039), temperature (21.7°C ± 0.25), salinity (15.44 PSU ± 0.18), dissolved oxygen (7.37 mg/L ± 0.21), and ammonia which was not detected throughout the exposures. The exposures lasted for 21-day at which time samples were collected, per U.S. EPA chronic testing protocols.

### 2.4 Behavior assay

At the end of the 21-day exposure period, a behavioral assay modified from (Siddiqui et al., 2022) was performed. Briefly, twelve well plates (Hutton et al., 2021), custom made with glass beakers, were randomly loaded with one fish and 8 mL of exposure solution per well. Fish were acclimated to the plate for at least 15 min and then placed into a DanioVision Observation Chamber (Noldus, Wageningen, the Netherlands). An additional 5 min of acclimation occurred inside the observation chamber in the dark followed by an alternating dark: light cycle with three 2-min periods of dark interspersed with 2-min of light. Data was binned by 30-s intervals. Total distance moved (TDM) (cm) and thigmotaxis, a measure of the organism’s closeness to the well wall and an indicator of anxiety, were the main behavioral endpoints. A total of 2-3 individuals from each replicate were assessed for behavioral effects. Behavioral tracking was conducted between 07:00 and 12:00 h, which encompassed the standard light period of the exposures. Behavior was recorded and tracked using a Basler Gen 1 Camera using Ethovision^®^ XT15 software, 1,280 × 960 resolution, 10,000 lux of light, and a 25/s frame rate.

### 2.5 Growth

Following the behavior assay, the organisms were euthanized humanly in accordance with OSU International Animal Care and Use protocol #0035 and placed in a 3% paraformaldehyde solution at 4°C until use. For growth index, 2 – 3 individuals from each replicate and treatment were imaged using an Olympus SZ61 Stereo Microscope and Olympus DP23 Microscope Digital Camera (Olympys Corporation, Tokyo, Japan), and length and width were measured using cellSens Imaging Software (Olympys Corporation, Tokyo, Japan). Growth index was modified from Siddiqui et al. with the following equation: $\frac{W}{L}\;x\;days$

Where *W* is width in cm, *L* is length in cm, and days is the length of the exposure (Siddiqui et al., 2023).

### 2.6 RNA-sequencing

Following 21-days of exposure to the control or MNP treatments, 2–4 Inland Silverside larvae per replicate (n = 3) were flash frozen in liquid nitrogen and stored at −80°C until use. Total RNA was extracted using RNeasy Mini Kits according to the manufacture’s recommendations (Qiagen, Hilden, Germany). Total RNA quality was checked with a BioAnalyzer 2,100 w/RNA nano chip (Agilent, Santa Clara, CA, United States) and quantified by Qubit 4 fluorometer (ThermoFisher, Waltham, MA United States) using the RNA-BR assay; RIN values were greater than 9. rRNA was removed using Illumina’s Ribo-Zero Plus kit (Illumina, San Diego, CA, United States) following standard directions in the protocol. rRNA depleted RNA was run on the BioAnalyzer 2,100 w/the RNA pico chip to verify the removal of rRNA. Libraries were than prepped with the Takara PrepX RNA-seq kit (Takara, San Jose, CA, United States) on the Apollo 324 robotic liquid handler. Final libraries were quantified by Qubit 4 fluorometer using the 1x dsDNA HS assay. Sizing of each library was checked on the TapeStation 4,200 w/the HS D5000 tape (Agilent, Santa Clara, CA, United States). All libraries were quantified by qPCR on the ABI 7500 fast instrument (ThermoFisher, Waltham, MA, United States) using KAPA library quantification kit (KAPA Biosystems, Wilmington, MA, United States). Libraries were normalized and pooled. Libraries were sequenced on the Illumina NextSeq 2000 instrument (Illumina, San Diego, CA, United States) as a 100 bp paired end P3 run.

### 2.7 Transcriptome assembly

Raw fastq data files were assessed for quality using FastQC. Adaptor sequences were removed from raw reads and reads were trimmed using cutadaptor. Filtered, pair end reads were used to assemble a *de novo* transcriptome using Trinity (v2.9.1) with default settings. Transcripts/unigenes were functionally annotated using Trinotate (v3.2.2). Briefly, transcripts/unigenes were subject to a homology search using BLASTX and sequence data from the Swissport database, protein domain identification (HMMER, v3.2.2), protein signal peptide and transmembrane domain prediction (signal, v4.1), and the annotation databases eggNOG, GO, and KEGG. Then, search results were loaded into the Trinotate SQLite database to create a whole annotation report.

### 2.8 Differentially expressed genes and functional enrichment analysis

Salmon was used to quantify gene-level count estimates of sequence reads against the *de novo* transcriptome. *DESeq2* was used to determine differentially expressed genes (DEGs). *P* values were adjusted using the Benjamani-Hochberg adjustment, and all expression values with an adjusted-p < 0.05 and |logFC| > 0 were considered differentially expressed. Enriched (*p* < 0.05) gene ontology (GO) terms were analyzed using *topGO* and KEGG functional analysis was performed using *clusterprofile.*

### 2.9 Statistical analysis

Statistical analysis was performed in R software version 4.2.2 (Vienna, Austria) and R Studio version 2022.12.0 + 353 (Boston, MA, United States). Growth data were normally distributed and were analyzed by one-way ANOVA followed by Tukey’s post-hoc test. Behavioral data were not normally distributed and were analyzed using Kruskal-Wallis’s test followed by Dunn’s test. Growth and behavioral results were considered statistically significant if *p* < 0.05.

## 3 Results and discussion

All particles generated expected spectra (Supplementary Figure S1). After 72 h of mechanical and solar weathering, there are no clear physical differences in particle appearance for tire particles (Supplementary Figures S2A, D, S3A, D) and PLA (Supplementary Figures S2B, E, S3B, E). However, the polyester microfibers were impacted (Supplementary Figures S2C, F, S3C, F). The polyester MFs that were not weathered appeared smooth and pristine. While, weathered polyester microfibers appear to have indentations and additional pieces attached to or fragmenting off of them.

The *de novo* transcriptome assembly resulted in 24,492 genes and a 98% mapping rate to all the genes in the fastq files. Inland Silverside survival throughout the exposures was above 70% for all treatments, which is expected for chronic exposures that begin with embryos (Hutton et al., 2024). Survival was as follows (mean ± standard error): control (80.4% ± 0.016), µTP (90.5% ± 0.095), µTP(W) (73.5% ± 0.082), nTP (71.2% ± 0.038), nTP (W) (71.5% ± 0.096), µPLA (75.5% ± 0.076), µPLA (W) (81.0% ± 0.13), nPLA (75.0% ± 0.072), nPLA (W) (84.3% ± 0.032), MF (79.2% ± 0.032), MF (W) (81.8% ± 0.091).

### 3.1 Effects on behavior and potential neurotoxicity

**FIGURE 1:**
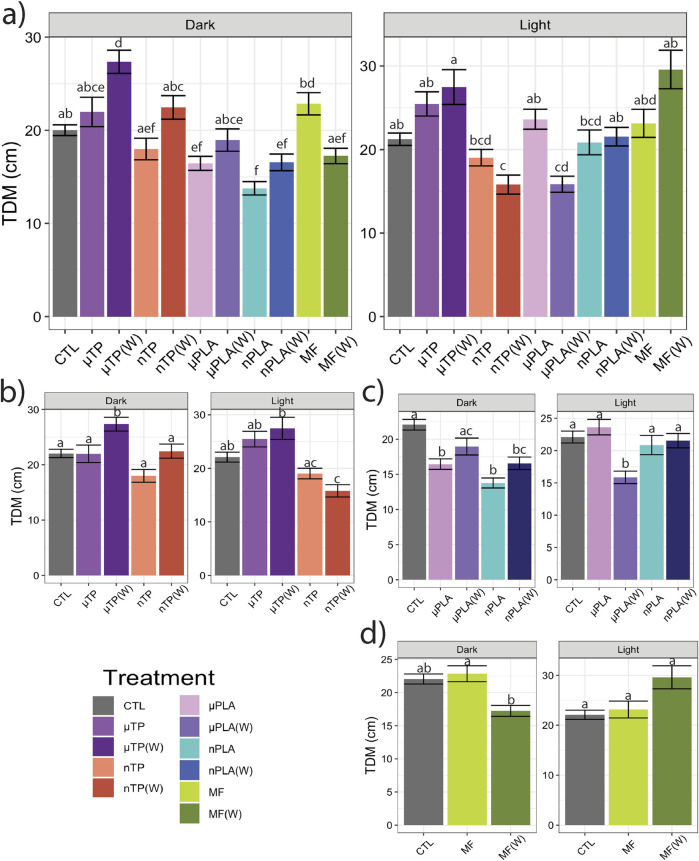
Total distance moved (TDM) of Inland Silverside larvae following 21-day exposure to **(A)** all the MNP treatments, **(B)** tire particle treatments, **(C)** PLA treatments, and **(D)** polyester MF treatments. TP, tire particle (exposed at 50 p/mL); PLA, polylactic acid (exposed at 50 p/mL); MF, polyester microfiber (exposed at 30 p/mL). W, weathered; particles were weathered under UV A, B, and C light on a shaker at 15 PSU to simulate wave action. Different letters represent statistically significant differences between treatments (p < 0.05, Tukey post-hoc test).

**FIGURE 2:**
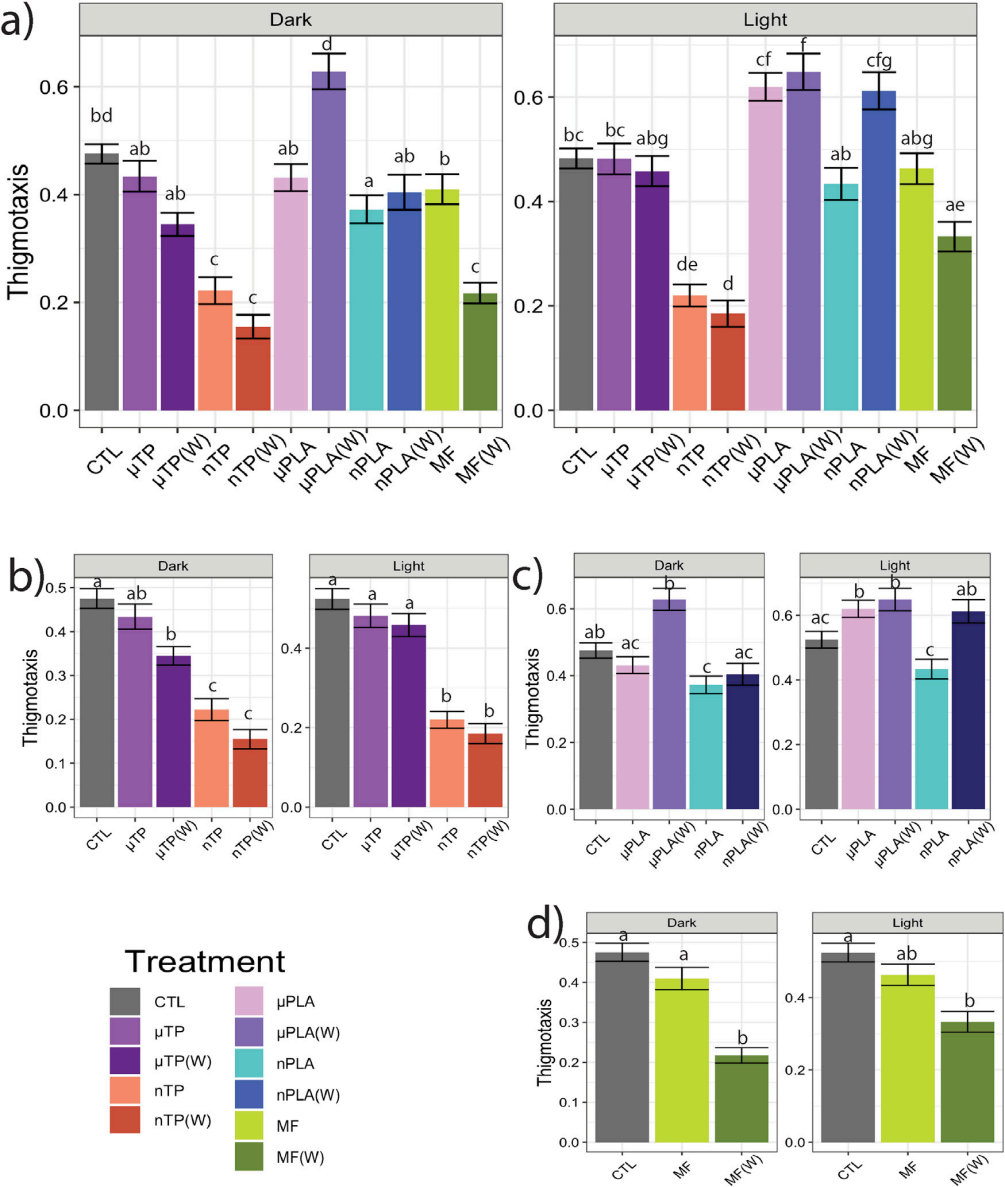
Thigmotaxis (wall hugging), a measure of anxiety-like behavior, in Inland Silverside larvae following 21-day exposure to **(A)** all the MNP treatments, **(B)** tire particle treatments, **(C)** PLA treatments, and **(D)** polyester MF treatments. TP, tire particle (exposed at 50 p/mL); PLA, polylactic acid (exposed at 50 p/mL); MF, polyester microfiber (exposed at 30 p/mL). W, weathered; particles were weathered under UV A, B, and C light on a shaker at 15 PSU to simulate wave action. Different letters represent statistically significant differences between treatments (*p* < 0.05, Tukey post-hoc test).

Following the 21-days of exposure to the MNP treatments, we assessed behavior of the Inland Silverside larvae and compared the changes in TDM, an indicator of exploration and activity, and thigmotaxis, an indicator of anxiety or boldness. Behavior is a common ecotoxicological endpoint that is frequently used to assess neurotoxicity but can also be an indicator of phenotypic, developmental effects. Exposure to µTP (W) induced hyperactivity (increased TDM) in the dark (Figures 1A, B) and nTP (W) and µPLA (W) exposure induced hypoactivity in the light (Figures 1A–C). µPLA, nPLA, nPLA (W), and MF (W) exposed Inland Silversides displayed hypoactivity in the dark but no effect on TDM was observed during the light cycle (Figures 1A, C, D). Exposure to µTP nTP, and MFs did not affect Inland Silverside larvae TDM. In terms of thigmotaxis (anxiety-like behavior), both nTP, nTP (W), and MF (W) decreased thigmotaxis in the dark and light (Figures 2A, B, D) and both µPLA and µPLA (W) showed increased thigmotaxis in the light while nPLA decreased thigmotaxis in the dark (Figures 2A, C). µTP, nPLA (W), and MF exposure did not affect thigmotaxis in Inland Silversides in this study.

Our lab has published behavioral data from acute studies of Inland Silverside larvae exposed to TPs (Siddiqui et al., 2022) and polyester MF (Siddiqui et al., 2023). When exposed to 50 p/mL of µTPs and nTPs at 15 PSU, 3-day post hatch Inland Silversides mainly displayed hypoactivity, except for µTP in the light which here hyperactive. Inland Silverside 3 dph larvae exposed to µTPs increased their time spent near the well wall (implying increased anxiety), while those exposed to nTPs increased their time in the center of the well (implying increased boldness) (Siddiqui et al., 2022). When exposed to 30 p/mL of polyester MFs at 15 PSU, 3-day post hatch Inland Silversides did not display a difference in TDM, similar to the present study, but did display decreased movement and a change in thigmotaxis in the dark. Zebrafish larvae exposed to µPLA fragments (∼2 µm in length) also displayed hypoactivity and increased thigmotaxis behavior (de Oliveira et al., 2021), similar to the fish in our study. In contrast, another behavior study with adult Zebrafish and PLA did not show changes in movement or anxiety index but did have significant differences in social behaviors that could have a meaningful impact on schooling fish (Chagas et al., 2021). The results from the acute exposures with Inland Silversides and those from the present study may differ due to age specific differences in response to MNPs or may be a result of the different lengths of exposure times.

All of the treatments from this study, except for nTPs, had a significant difference in fish behavior between the unweathered and weathered particles in at least one behavioral endpoint. In addition, the growth index from unweathered nTP and unweathered polyester MF treatments were significantly decreased relative to their respective weathered particles (p < 0.05, Tukey post-hoc test) (Supplementary Figure S4). In a study with the Gilt-head bream, *Sparus aurata*, low density polyethylene, both virgin and weathered fragments (100–500 µm in length), reduced avoidance behavior, suggesting increased boldness, but did not affect any of the other behavior endpoints, such as freezing, chasing, moving, or feeding behaviors (Rios-Fuster et al., 2021). A chronic study of environmental microplastics fed to larval Japanese Medaka, *Oryzias latipes,* found hyperactivity (Pannetier et al., 2020). More studies are needed to better understand what characteristics of microplastics and associated chemicals, which may have played a role in our study since only unweathered particles affected growth, are important in influencing toxicity.

Both the chemical and physical impacts of MNPs must be considered when examining their potential effects on behavior. Specifically, whether the physical presence of MNPs alters behavior through the organism’s ability to sense the particles in the water. In our study, the organisms were kept in their exposure solutions during the behavioral assay, meaning we would be able to detect differences in behavior caused by the organism’s interaction with the particle. µPLA and µPLA(W) each had one instance of hypoactivity but neither µTP, µTP(W), MF, nor MF(W) affected TDM. This indicates that presence of the larger particles did not have a substantial effect on TDM and that the fish were not influenced by their potential presence in the water. Therefore, it is most likely that the MNPs affected behavior through mechanisms related to development and neurotoxicity. Toxicity may be a result of a chemical found on the particle or its leachate, but it may also be due to physical interactions of nanoplastics with tissues and cells (Thornton Hampton et al., 2022).

It has been well documented that nanoplastics, and other nanoparticles, can cross the blood-brain barrier (Kashiwada, 2006), in fact, some nanoplastics, including PLA based particles, are purposefully engineered to cross the blood-brain barrier for drug delivery (Zhou et al., 2018). Therefore, it is highly possible the nanoplastics from this study were able to enter the brains of the Inland Silverside larvae and affect brain function. Nanoplastic exposure has been demonstrated to reduce AChE activity, alter gene expression in the brain, and induce oxidative stress (Torres-Ruiz et al., 2023). It is likely that nanoplastic exposure in our study induced changes in behavior, in part, due to neurotoxicity.

### 3.2 Microplastics affect gene regulation

**FIGURE 3:**
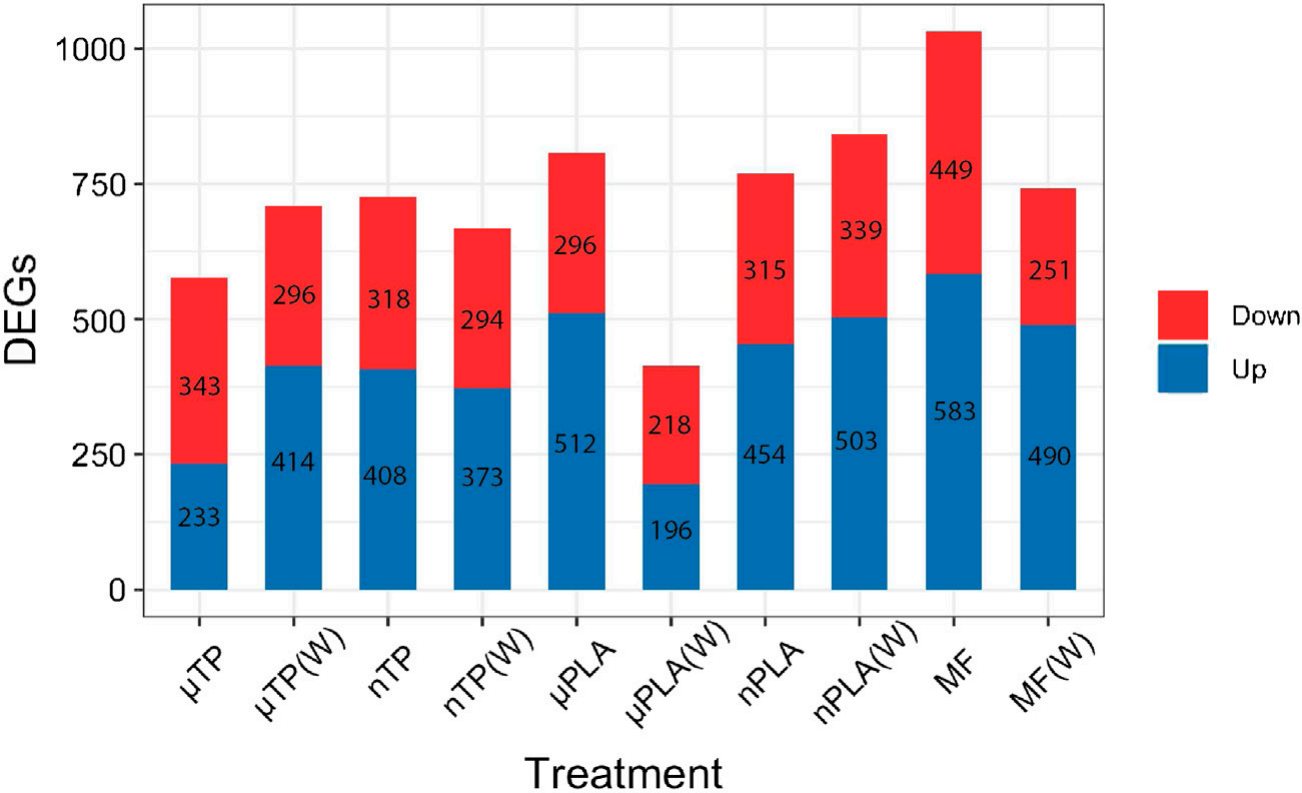
Bar chart of the number of differentially expressed genes in Inland Silverside larvae following 21-day exposure to all MNP treatments. TP, tire particle (exposed at 50 p/mL); PLA, polylactic acid (exposed at 50 p/mL); MF, polyester microfiber (exposed at 30 p/mL). W, weathered; particles were weathered under UV A, B, and C light on a shaker at 15 PSU to simulate wave action. Downregulated genes are on top (red) and upregulated genes are on the bottom (blue).

Using RNA sequencing we investigated the effects of MNP exposure on differential gene expression. We compare enrichment of gene ontology and KEGG pathways between up and downregulated genes. Overall, there were 2,389 unique differentially expressed genes (DEGs) across all treatments (Supplementary Figure S5). Polyester MF caused the most DEGs in all the treatments and µPLA (W) caused the fewest DEGs (Figure 3). Hierarchically clustering performed with the control and all ten treatments did not result in distinctive clusters between treatments other than the controls, MF, and µTP(W) (Supplementary Figure S6A). When hierarchical clustering was done between the control and just one treatment, the treatments always clustered away from the control (Supplementary Figures S6B–K). This suggests that the individual effects of microplastics compared to the control are distinct but the effects of MNP exposure are not distinct enough to have separate clusters of the MNP treatments. It is still unclear from the literature how important polymer type is in driving toxicity. Some studies have observed differential effects while others do not see differences in toxicity between multiple polymer types (Thornton Hampton et al., 2022). The overwhelming bias in the literature for polystyrene-based exposures makes it challenging to know given the current data if polymer type is an important factor in MNP toxicity.

Biological process (BP), molecular function (MF), and cellular component (CC) gene ontology were conducted for each treatment, keeping up and downregulated genes separate. The significantly enriched GO terms were compiled, and the top 15 positively and negatively enriched GO terms shared from all treatments were compared visually. nTP, nPLA (W), and MF (W) significant upregulated DEGs were enrichment of BP terms related to regulation of protein and peptide secretion (GO: 0050714 and GO: 0002793). nPLA (W) and MF (W) significantly upregulated DEGs also included enrichment of BPs pathway related to protein kinase signaling (GO:0010737). µPLA, nTP (W), and nTP downregulated DEGs were enriched for the BP term cellular protein metabolic process (GO: 0032268), further suggesting MNP exposure affected protein metabolism and maintenance. The specific mechanism by which protein metabolism is disrupted may differ depending on the MNP type, which would explain why the different MNPs show responses in different pathways and directions.

**FIGURE 4:**
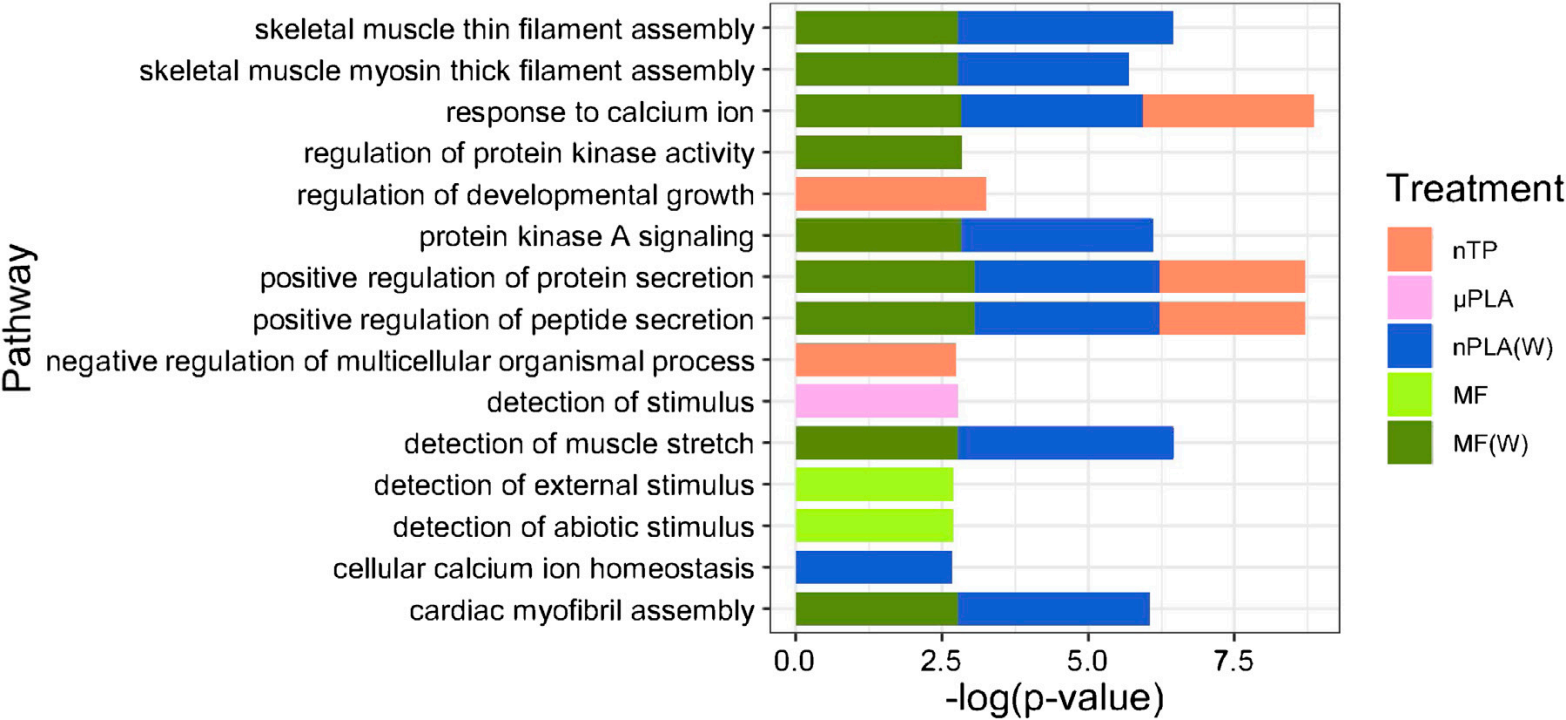
Stacked bar plot showing the top 15 upregulated GO biological process terms in Inland Silverside larvae following 21-day exposure to the MNP treatments. Not all treatments are displayed as they were not represented by the top 15 GO terms. TP, tire particle (exposed at 50 p/mL); PLA, polylactic acid (exposed at 50 p/mL); MF, polyester microfiber (exposed at 30 p/mL). W, weathered; particles were weathered under UV A, B, and C light on a shaker at 15 PSU to simulate wave action. p < 0.05, logFC > 0.

**FIGURE 5:**
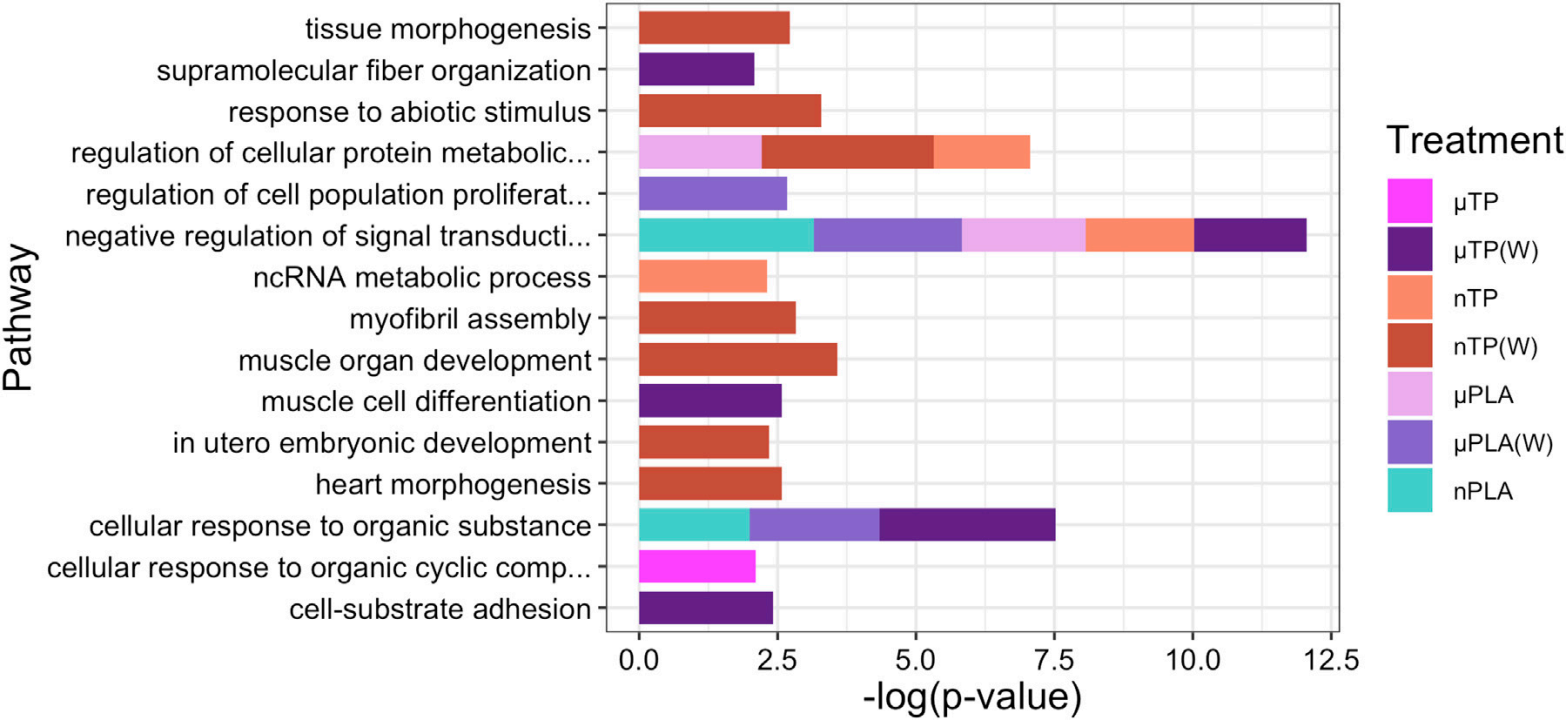
Stacked bar plot showing the top 15 downregulated GO biological process terms in Inland Silverside larvae following 21-day exposure to all the MNP treatments. Not all treatments are displayed as they were not represented by the top 15 GO terms. TP, tire particle (exposed at 50 p/mL); PLA, polylactic acid (exposed at 50 p/mL); MF, polyester microfiber (exposed at 30 p/mL). W, weathered; particles were weathered under UV A, B, and C light on a shaker at 15 PSU to simulate wave action. p < 0.05, logFC < 0.

**FIGURE 6:**
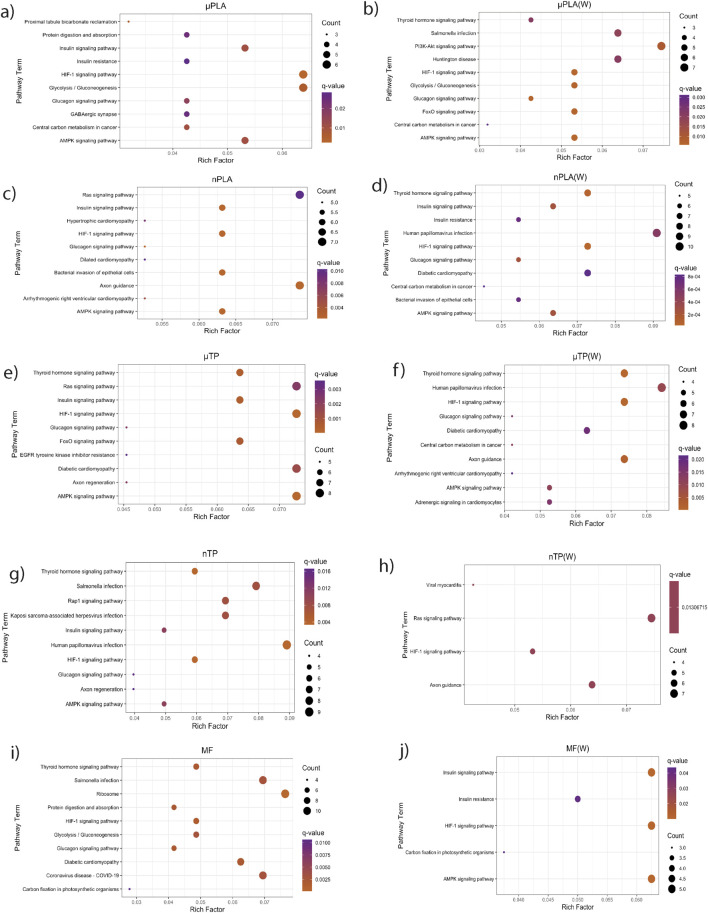
KEGG pathways enriched from downregulated genes in Inland Silverside larvae following 21-day exposure to all the MNP treatments. TP, tire particle (exposed at 50 p/mL); PLA, polylactic acid (exposed at 50 p/mL); MF, polyester microfiber (exposed at 30 p/mL). W, weathered; particles were weathered under UV A, B, and C light on a shaker at 15 PSU to simulate wave action. p < 0.05, logFC < 0.

While Inland Silversides are able to withstand a wide range of salinities, changes in ion regulation and other cellular processes can affect osmoregulation. Polystyrene microspheres altered osmoregulation in mussels following exposure at 50 p/mL (Cappello et al., 2021), similar to the concentrations used in the present study. Here, nTP, nPLA (W), and MF (W) upregulated DEGs were enriched for the BP term calcium ions (GO: 0051592), which was found in fish responding to high salinity stress (Figure 4). In addition, nPLA (W) upregulated DEGs were enriched for cellular calcium ion homeostasis (GO:0006874), which may be indicative of the organism’s ability to withstand the effects of nPLA (W) exposure on ionic balance. nPLA, µPLA (W), µPLA, nTP, and µTP (W) downregulated DEGs were enriched for negative regulation of signal transduction (GO:0009968) (Figure 5). Signal transduction pathways are extremely important in osmoregulation and osmosensing (Evans, 2010). In fact, it is theorized that signal transduction events are critical to the success of euryhaline species and their ability to tolerate osmotic stress (Evans, 2010). Additionally, HF-1 signal transduction KEGG pathway was decreased for all MNP exposures (Figures 6A–J). A number of other signaling KEGG pathways were increased following nPLA (W), µTP (W), nTP, nTP (W), MF, and MF (W) exposure such as HIF-1 and insulin signaling pathways, as well as others (Supplementary Figure S7). A decrease in signal transduction pathways could impact the fish’s ability to tolerate changes in salinity and depending on the severity of the reduction could have a meaningful impactful on their osmoregulatory capabilities, as well as their capacity to deal with other environmental stressors (DeCourten et al., 2019; Major et al., 2020). HIF-1, or hypoxia-induction factor, signaling pathway is involved in the organism’s response to hypoxia, combined exposure to hypoxia and MNPs exacerbates effects (Li et al., 2021). Additional studies are needed to confirm this hypothesis, future studies could look at the effects of MNPs on osmoregulation of euryhaline fish.

Both nPLA (W) and MF (W) had increased enrichment of BP pathways related to muscle filament assembly (GO:0030240/GO:0030241), muscle stretch (GO:0035995), and cardiac myofibril assembly (GO:0055003) (Figure 4). Further, analysis of gene ontology terms from the CC pathways shows that nTP, nTP (W), nPLA (W), and MF (W) had an upregulation of the M band (GO:0031430), nTP, nPLA (W), and MF (W) had an upregulation of striated muscle thin filament, and nTP, nPLA (W), MF, and MF (W) upregulated genes disrupted to titin-telethonin complex (GO:1990733) which are both related to Z disk function (Supplementary Figure S10). µPLA, nTP, and MF had increased enrichment of Z-disk (Supplementary Figure S10) and nTP(W) had decreased pathways related to the Z-disk (GO:0030018) (Supplementary Figure S10). Additionally, myofibril (GO:0030016) and actin cytoskeleton (GO:0015629) were downregulated in µPLA and nTP (W) and µPLA (W), respectively and are both also related to skeletal and cardiac muscle function (Supplementary Figure S11). The M band and Z disk are a part of the sarcomere and are crucial for muscle contraction (Zou et al., 2006; Lange et al., 2020). They are involved in both skeletal and cardiac muscle contraction and disruptions to their function are associated with various muscular diseases (Selcen and Carpén, 2008; Lange et al., 2020). While not in the top 15 significantly upregulated enriched BP pathways, nTP, nPLA (W), and MF (W) all had an increase in the sarcomerogenesis pathway (GO:0048769) and nTP, nPLA, nPLA (W), MF, and MF (W) all had an increase in sarcomere organization (GO:0045214) (Supplementary Table S1). There were also a number of pathways disrupted for BP terms related to muscle function that were not in the top 15 affected pathways. For example, muscle contraction (GO:0006936), cardiac muscle contraction (GO:0060048), and heart contraction (GO:0060047) were upregulated for BP terms (Supplementary Table S1) and striated muscle contraction (GO:0006941), muscle organ development (GO:0007517) and muscle cell differentiation (GO:0042692) were downregulated (Supplementary Table S2). Disruption of GO terms pathways related to skeletal and cardiac muscle function strongly suggests that the MNPs had an effect on muscle function, likely through dysregulation of sarcomeres. We also found evidence of disrupted KEGG pathways related to cardiac function which could be a result of effects on cardiac muscle function (Figures 6C–F, H, I).

Overall, our findings and those of others strongly suggest that MNPs have the potential to disrupt muscle function, at least at a cellular and molecular level (Han et al., 2021; Shengchen et al., 2021; Wang et al., 2021; Xu et al., 2024; Zhong et al., 2024). It is unclear how these molecular effects on sarcomeres and muscle function may manifest in more apical endpoints. For example, there does not appear to be an association between the treatments mentioned above and impacts to TDM (Figures 1A–D). While nTP, nTP (W), nPLA, and MF (W), do result in decreased thigmotaxis, it is more likely that changes in thigmotaxis are related to neurotoxicity pathways and not muscle development. Changes in muscle development and function are more likely to affect movement and not location preference. It is possible that the negative effects of dysregulated molecular pathways will become apparent later in life since both M-band and Z-disk dysregulation are involved in muscle disease (Lange et al., 2020; Luther, 2009). However, future MNPs studies with endpoints targeting muscular development and function would be needed to confirm this hypothesis, studies examining muscle cellular pathology would provide insight into potential muscular disease.

Finally, we observed dysregulation of BP terms related to sensory detection. µPLA exposure upregulated detection of stimulus (GO:0051606), unweathered MF exposure upregulated detection of external stimulus (GO:0009581) and detection of abiotic stimulus (GO:0009582), and nTP(W) exposure downregulated detection of abiotic stimulus (GO:0009582). Similar effects on sensory detection were also observed in coral (Bove et al., 2022) but this is the first time this effect has been reported in fish. Disruption of these pathways may indicate a neurotoxic effect and could affect behavior, predator - prey interactions, or the fish’s ability to interact with its environment. As discussed above, we did not find evidence to suggest the presence of the MNPs in the behavior wells influenced TDM in this study. In order to better understand if these dysregulated pathways impact fish apically, future studies should focus on endpoints related to detection of predators, changes to schooling structure, or ability to detect changes in water quality (i.e., detection of pollution, changes in temperature, dissolved oxygen, or water flow) (Dasgupta et al., 2022; Fréon et al., 1992).

### 3.3 Weathered microfibers show similar effects as some nanoplastics

Microfibers enter the aquatic environment from sources such as ropes, fishing nets, and textiles and are often the dominant particle type found in estuarine and marine samples (Granek et al., 2022; Boisen et al., 2024), make up to 96% of particles found in one estuary, 80% of which were synthetic (Bessa et al., 2018). Domestic laundering has been identified as not only a prominent source of MFs but also one of the primary sources for synthetic microfibers (Galvão et al., 2020). It is especially important to consider the effects of UV-C photodegradation on synthetic microfibers as they are often found in wastewater from laundering where they may be treated UV-C at WWTPs, which enhances their degradation (Easton et al., 2023) and after-which they enter the environment via effluent, or more commonly due to biosolid application and subsequent run-off (Geyer et al., 2022).

The only treatment to have a significant effect on growth was unweathered polyester MF’s (p < 0.05, Tukey post-hoc test) (Supplementary Figure S4). All other treatments were unchanged relative to the control (p > 0.05, Tukey post-hoc test). The reduction in growth may be due to false satiation (fullness) or blockage of the gut or digestive tract (de Sá et al., 2015; Gao et al., 2022). Fiber bundles are defined as a mass of more than 20 fibers that cannot be untangled (Rochman, 2015), and there is some evidence to suggest that fibers are more difficult to excrete (Granek et al., 2022). Our exposure concentration of 30 p/mL would result in approximately 15,000 particles per replicate (500 mL solution); therefore, it is possible that some portion of those fibers formed fiber bundles that were unintentionally ingested and altered larval feeding behavior.

It has previously been demonstrated that plastic exposed to UV weathering conditions will breakdown into nanoplastics (Song et al., 2020). In a study of polyester microfiber degradation, it was found that exposure to UV C radiation caused the fibers to lose mass, however, microfibers are not likely to degrade into a fiber-like shape but are more likely to be in a fragment shape (Easton et al., 2023). SEM images of weathered polyester microfiber used in this study showed fragmentation of the microfiber (Supplementary Figure S2F, S3F). Compared to natural fibers, polyester creates more fragments than wool but releases fewer chemicals and additives when undergoing UV-A and B radiation (Sørensen et al., 2021). The weathering process likely caused the polyester MF in our study to become more brittle and degradable, creating smaller, possibly nanosized particles. Under this scenario, the unweathered particles, which showed no signs of weathering (Supplementary Figure S2C, S3C), may have taken up more space inside the gut, creating larger blockages, which impaired feeding and reduced growth. It is also possible that the new polyester MFs had more chemicals associated with them compared to the weathered MFs. When exposed to the same concentration of polyester MFs for 96 h, Inland Silversides did not demonstrate decreased growth (Siddiqui et al., 2023). However, the fish here were exposed throughout a critical growth stage during which the fish begin to eat brine shrimp, allowing them to ingest larger fibers or possible fiber bundles.

Overall, MF (W)s caused similar effects as other nanosized particles; notably, and as discussed above, the MF (W), nTP, nTP (W), and nPLA treatments all demonstrated decreased thigmotaxis, an indicator of increased boldness. Interestingly, MF (W) and nPLA (W) both caused upregulation of similar GO terms related to muscle function and development (Figure 3). The similar responses observed between the nanoparticles and MF(W) as well as the visual confirmation of MF particle weathering (Supplementary Figure S2F, S3F), suggests nano-sized polyester particles were generated during the MF weathering process. However, whether nanosized particles all induced similar mechanisms of toxicity is unclear. MF (W), nPLA (W), and nTP induced similar mechanistic effects but nPLA (W) shared no behavioral effects with any of the other nanosized treatments. More in-depth studies into neurological or physiological pathways involving muscle development and function would be needed to determine if nanosized particles consistently induce similar mechanisms of toxicity.

### 3.4 Implications for the environment

The proprietary nature of many consumer plastic materials makes comparison of toxicity testing from in-house generated particles challenging as different chemical mixtures and manufacturing protocols could cause different toxicity profiles (Chibwe et al., 2022). One of the limitations of this study is the use of in-house particles, which may represent a unique set of size distributions and leachate composition. Consequently, understanding these variations is crucial for accurately assessing environmental hazards. While studies using polystyrene microspheres are not considered environmentally relevant due to the lack of representation of commonly found particles, they do have the benefit of being relatively standardized across studies.

Concentrations of microplastics in the estuarine/marine environment vary from 4.0 × 10^−5^ p/L to 3.0 × 10^−3^ p/L (Markic et al., 2022), storm water runoff has some of the highest reported concentrations of microplastics with estimates of 0.1–10,000 p/L (Österlund et al., 2023). However, evidence suggests that current estimates of environmental concentrations, especially for smaller particles, are inaccurate and likely a vast underestimate (Conkle et al., 2018; Cunningham et al., 2023). The concentrations used here do not represent concentrations currently found in the aquatic environment (Kashiwabara et al., 2021; Zhao et al., 2024). As discussed above, current technology cannot reliably quantify nanoparticles in the environment. Occurrence studies have found that the smallest sizes sampled overwhelmingly have the greatest percentage of particles (Du et al., 2024), which indicates that smaller micro sized, and nano sized plastic particles likely exist in higher concentrations that what is currently estimated. In addition, while these concentrations may not represent current MNP contamination, plastic production is growing and pollution is worsening, it is possible these concentrations may be environmentally relevant in the future.

## 4 Conclusion

Microplastics are ubiquitous throughout the aquatic environment and have become a contaminant of emerging concern, drawing the attention of scientists, policymakers, and the public; and they are now the focus of global treaty currently under negotiation (Brander et al., 2024b). In this study we sought to better understand potential mechanisms of toxicity of environmentally relevant particle types and sizes. We saw effects on growth from microfibers and impacts on behavior and anxiety from a number of our tested particles. Gene expression data suggests that muscle contraction and function may be dysregulated by MNP exposure as well as cell signaling and osmoregulation. Weathered particles did not necessarily increase toxicity, as has been seen in some studies. However, we did find that weathering can alter the mechanism of toxicity, particularly for microfibers. Although the effects found in this study may represent a hazard, it is not clear what the risk of these outcomes are to environmental fish populations. The concentrations used in this study are higher than those currently detected in the environment and were selected for the specific purpose of identifying potential mechanisms of toxicity. Future studies with more targeted endpoints like osmoregulation, muscle function, growth, and others found in this work, along with additional concentrations for dose-response curves would be beneficial to risk assessment.

## Data Availability

The datasets presented in this study can be found in online repositories. The names of the repository/repositories and accession number(s) can be found below: https://www.ncbi.nlm.nih.gov/, PRJNA1176563.
