# Supplementary material for: Behavioral and molecular effects of micro and nanoplastics across three plastic types in fish: weathered microfibers induce a similar response to nanosized particles
Source: Front Toxicol. 2024 Nov 26;6:1490223. doi: 10.3389/ftox.2024.1490223 (PMC11628497; doi:10.3389/ftox.2024.1490223)
Supplement: Supplementary file 1 [file Table1.docx]

GROWTH, BEHAVIOR, AND GENE EXPRESSION CHANGES ACROSS MICRO AND NANO PLASTIC SHAPES, SIZES, AND WEATHERING CONDITIONS IN A MODEL FISH

Sara J. Hutton^1^, Lauren Kashiwabara^2^, Erin Anderson^1^, Samreen Siddiqui^2^, Bryan Harper^1^, Stacey Harper^1,3^, and Susanne M. Brander^2^

1. Oregon State University, Department of Environmental and Molecular Toxicology, Corvallis OR
2. Oregon State University, Fisheries, Wildlife, and Conservation Sciences Department Coastal Oregon Marine Experiment Station, Hatfield Marine Science Center
3. Oregon State University, School of Chemical, Biological and Environmental Engineering, Corvallis OR

a.

c.

b.


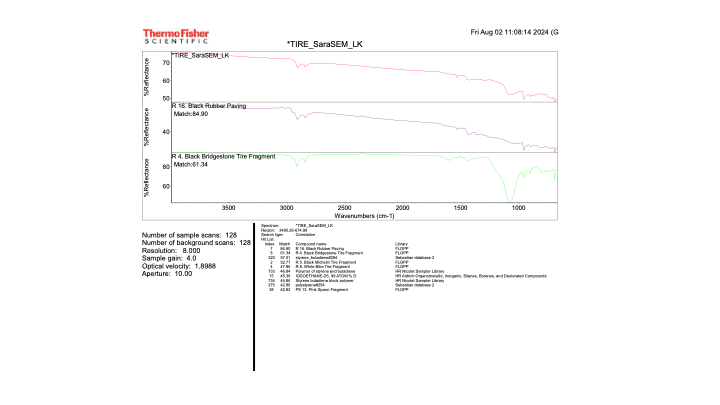

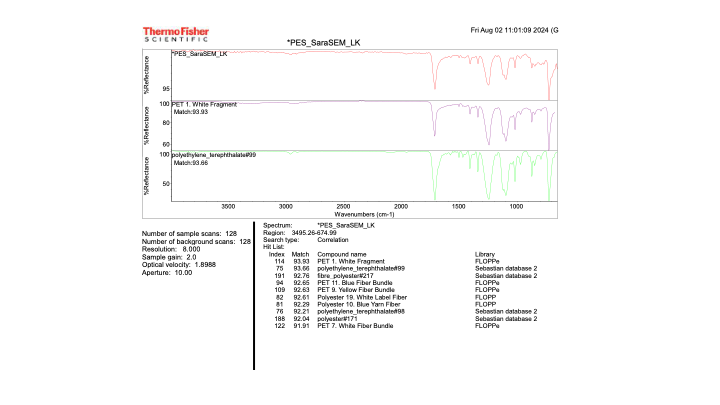

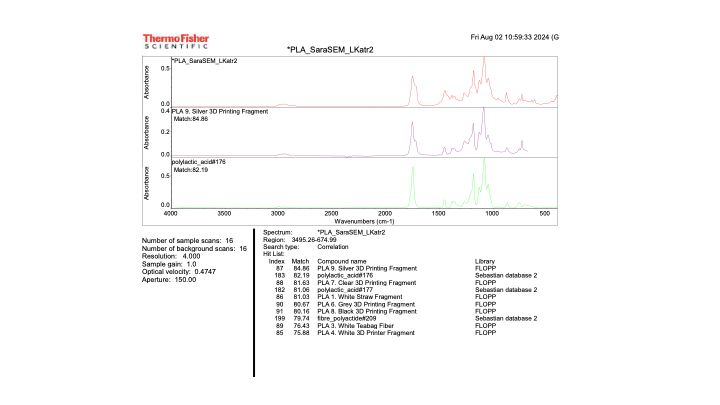


**Figure S1.** FTIR spectra from non-weathered (a) tire, (b) PLA, and (c) polyester fiberS

**
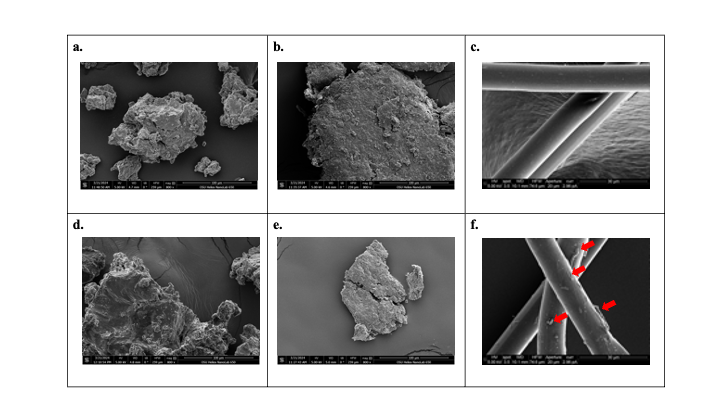
Figure S2.** Non-weathered (a) tire, (b) PLA, and (c) polyester fiber and weathered (d) tire, (e) PLA, and (f) polyester fiber images taken on SEM at lower magnification (800x for tire/PLA 2,000x for fibers). Arrows indicate results from weathering.


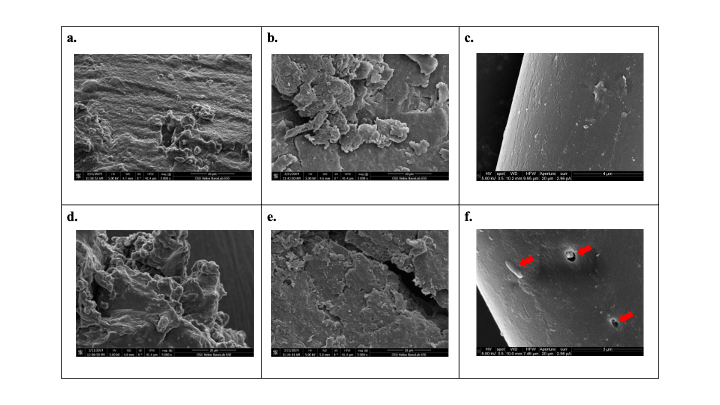
**Figure S3.** Non-weathered (a) tire, (b) PLA, and (c) polyester fiber and weathered (d) tire, (e) PLA, and (f) polyester fiber images taken on SEM at higher magnification (5000x for tire/PLA (a, b, d, e) and 15,000x (c) and 20,000x (f) for fibers). Arrows indicate results from weathering.

**Figure 4.** Boxplot of Inland Silverside growth index (width/length) after 21-day exposure to control and the ten treatmentS µTP, µTP(W), nTP, nTP(W), µPLA, µPLA(W), nPLA, nPLA(W) exposures contained 50 particles/mL and MF & MF(W) contained 30 particles/mL. TP = tire particle, PLA = polylactic acid, MF = polyester microfiber, W = weathered. Different letters denote p < 0.05 relative to all treatmentS One-way ANOVA followed by Tukey post-hoc test.


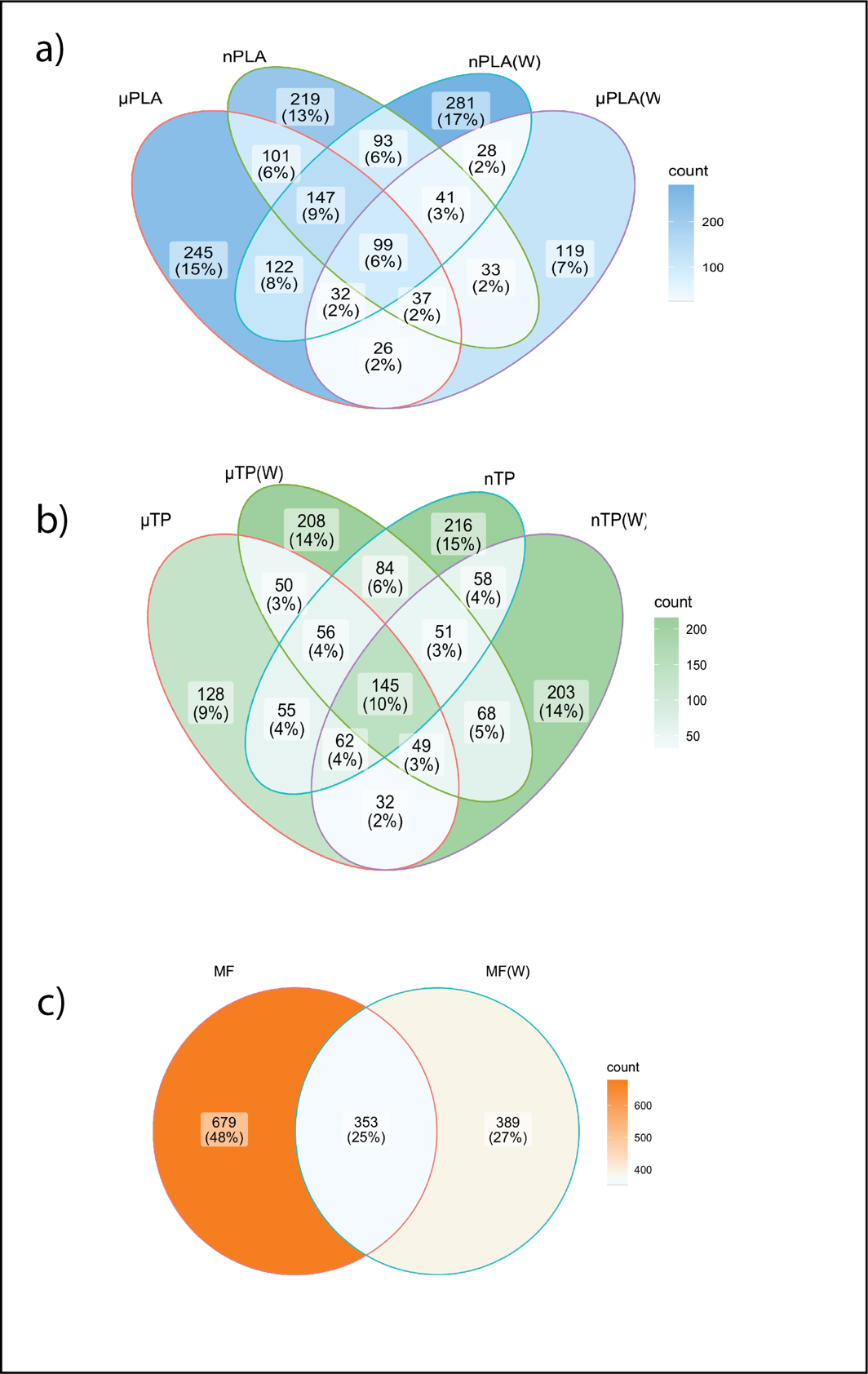


**Figure S5.** Venn diagram of the number of differentially expressed genes in Inland Silverside larvae after 21-day exposure to the control and the ten treatmentS µTP, µTP(W), nTP, nTP(W), µPLA, µPLA(W), nPLA, nPLA(W) exposures contained 50 particles/mL and MF & MF(W) contained 30 particles/mL. TP = tire particle, PLA = polylactic acid, MF = polyester microfiber, W = weathered.

**Figure S6.** Hierarchical clustering of differentially expressed genes in Inland Silverside larvae after 21-day exposure to a) the control and all the ten treatments, b) control vs µPLA, c) control vs µPLA(W) , d) control vs nPLA, , e) control vs nPLA(W), f) control vs µTP, g) control vs µTP(W) , h) control vs nTP, i) control vs nTP(W) , j) control vs MF, i) control vs MF(W). TP = tire particle, PLA = polylactic acid, MF = polyester microfiber, W = weathered.


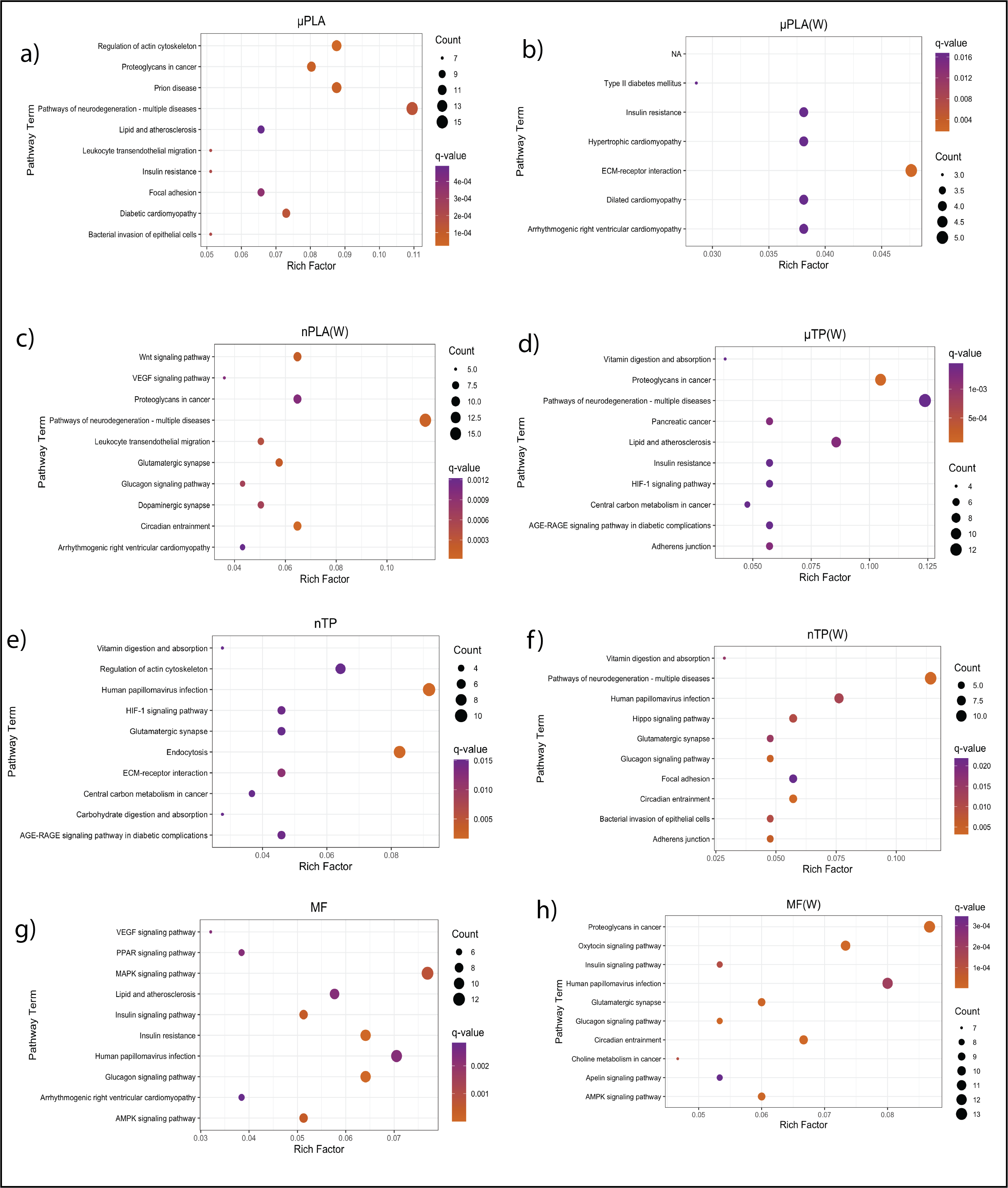


**Figure S7.** KEGG pathways enriched from downregulated genes in Inland Silverside larvae following 21-day exposure to all the MNP treatmentS TP = tire particle (exposed at 50 p/mL); PLA = polylactic acid (exposed at 50 p/mL); MF = polyester microfiber (exposed at 30 p/mL). W = weathered; particles were weathered under UV A, B, and C light on a shaker at 15 PSU to simulate wave action. p < 0.05, logFC > 0. nPLA and µTP did not have upregulated KEGG pathwayS

**Table S1.** Table of gene ontology biological process terms from upregulated genes from Inland Silverside larvae after 21-day exposure to the control and the ten treatmentS µTP, µTP(W), nTP, nTP(W), µPLA, µPLA(W), nPLA, nPLA(W) exposures contained 50 particles/mL and MF & MF(W) contained 30 particles/mL. TP = tire particle, PLA = polylactic acid, MF = polyester microfiber, W = weathered.

|  | **GO.ID** | **Term** | **classicKS** | **treatment** |
| --- | --- | --- | --- | --- |
| **1** | GO:0051606 | detection of stimulus | 0.0017 | µPLA |
| **2** | GO:0060047 | heart contraction | 0.0105 | µPLA |
| **3** | GO:0006936 | muscle contraction | 0.0130 | µPLA |
| **4** | GO:0048729 | tissue morphogenesis | 0.0025 | µPLA |
| **5** | GO:0006417 | regulation of translation | 0.0268 | µPLA |
| **6** | GO:0048646 | anatomical structure formation involved in morphogenesis | 0.0274 | µPLA |
| **7** | GO:0055001 | muscle cell development | 0.0279 | µPLA |
| **8** | GO:0008361 | regulation of cell size | 0.0305 | µPLA |
| **9** | GO:0044089 | positive regulation of cellular component biogenesis | 0.0356 | µPLA |
| **10** | GO:0033044 | regulation of chromosome organization | 0.0360 | µPLA |
| **11** | GO:0010038 | response to metal ion | 0.0115 | nPLA |
| **12** | GO:0098771 | inorganic ion homeostasis | 0.0461 | nPLA |
| **13** | GO:0016055 | Wnt signaling pathway | 0.0055 | nPLA |
| **14** | GO:0045214 | sarcomere organization | 0.0140 | nPLA |
| **15** | GO:0007420 | brain development | 0.0150 | nPLA |
| **16** | GO:0000226 | microtubule cytoskeleton organization | 0.0150 | nPLA |
| **17** | GO:0001667 | ameboidal-type cell migration | 0.0164 | nPLA |
| **18** | GO:0048769 | sarcomerogenesis | 0.00021 | nPLA(W) |
| **19** | GO:0035995 | detection of muscle stretch | 0.00021 | nPLA(W) |
| **20** | GO:0030240 | skeletal muscle thin filament assembly | 0.00021 | nPLA(W) |
| **21** | GO:0055003 | cardiac myofibril assembly | 0.00053 | nPLA(W) |
| **22** | GO:0010737 | protein kinase A signaling | 0.00053 | nPLA(W) |
| **23** | GO:0050714 | positive regulation of protein secretion | 0.00070 | nPLA(W) |
| **24** | GO:0002793 | positive regulation of peptide secretion | 0.00070 | nPLA(W) |
| **25** | GO:0051592 | response to calcium ion | 0.00079 | nPLA(W) |
| **26** | GO:0060048 | cardiac muscle contraction | 0.00100 | nPLA(W) |
| **27** | GO:0045214 | sarcomere organization | 0.00120 | nPLA(W) |
| **28** | GO:0030241 | skeletal muscle myosin thick filament assembly | 0.00120 | nPLA(W) |
| **29** | GO:0055008 | cardiac muscle tissue morphogenesis | 0.00190 | nPLA(W) |
| **30** | GO:0006874 | cellular calcium ion homeostasis | 0.00211 | nPLA(W) |
| **31** | GO:0043056 | forward locomotion | 0.00548 | nPLA(W) |
| **32** | GO:0001756 | somitogenesis | 0.00548 | nPLA(W) |
| **33** | GO:0021591 | ventricular system development | 0.00548 | nPLA(W) |
| **34** | GO:0001701 | in utero embryonic development | 0.00614 | nPLA(W) |
| **35** | GO:0006936 | muscle contraction | 0.00219 | nPLA(W) |
| **36** | GO:0048583 | regulation of response to stimulus | 0.0233 | µPLA(W) |
| **37** | GO:0022607 | cellular component assembly | 0.0254 | µPLA(W) |
| **38** | GO:0097435 | supramolecular fiber organization | 0.0064 | µPLA(W) |
| **39** | GO:0010038 | response to metal ion | 0.039 | µTP |
| **40** | GO:0030239 | myofibril assembly | 0.015 | µTP |
| **41** | GO:0045087 | innate immune response | 0.026 | µTP |
| **42** | GO:0015833 | peptide transport | 0.027 | µTP |
| **43** | GO:0044260 | cellular macromolecule metabolic process | 0.0228 | µTP(W) |
| **44** | GO:1902275 | regulation of chromatin organization | 0.0044 | µTP(W) |
| **45** | GO:0048585 | negative regulation of response to stimulus | 0.0163 | µTP(W) |
| **46** | GO:0031056 | regulation of histone modification | 0.0082 | µTP(W) |
| **47** | GO:0006357 | regulation of transcription by RNA polymerase II | 0.0142 | µTP(W) |
| **48** | GO:0002684 | positive regulation of immune system process | 0.0145 | µTP(W) |
| **49** | GO:0010628 | positive regulation of gene expression | 0.0145 | µTP(W) |
| **50** | GO:0071375 | cellular response to peptide hormone sti... | 0.0170 | µTP(W) |
| **51** | GO:0048514 | blood vessel morphogenesis | 0.0429 | µTP(W) |
| **52** | GO:0048638 | regulation of developmental growth | 0.00056 | nTP |
| **53** | GO:0051592 | response to calcium ion | 0.00116 | nTP |
| **54** | GO:0045214 | sarcomere organization | 0.00116 | nTP |
| **55** | GO:0051241 | negative regulation of multicellular organismal process | 0.00180 | nTP |
| **56** | GO:0010628 | positive regulation of gene expression | 0.00192 | nTP |
| **57** | GO:0098662 | inorganic cation transmembrane transport | 0.00252 | nTP |
| **58** | GO:0016049 | cell growth | 0.00264 | nTP |
| **59** | GO:0002793 | positive regulation of peptide secretion | 0.00315 | nTP |
| **60** | GO:0050714 | positive regulation of protein secretion | 0.00315 | nTP |
| **61** | GO:0007420 | brain development | 0.00380 | nTP |
| **62** | GO:0060048 | cardiac muscle contraction | 0.00527 | nTP |
| **63** | GO:0010942 | positive regulation of cell death | 0.02351 | nTP |
| **64** | GO:0006936 | muscle contraction | 0.01817 | nTP |
| **65** | GO:0048769 | sarcomerogenesis | 0.00600 | nTP |
| **66** | GO:0055008 | cardiac muscle tissue morphogenesis | 0.00600 | nTP |
| **67** | GO:0003007 | heart morphogenesis | 0.0210 | nTP(W) |
| **68** | GO:0006936 | muscle contraction | 0.0022 | nTP(W) |
| **69** | GO:0048878 | chemical homeostasis | 0.0189 | MF |
| **70** | GO:0009749 | response to glucose | 0.0466 | MF |
| **71** | GO:0003300 | cardiac muscle hypertrophy | 0.0050 | MF |
| **72** | GO:0045214 | sarcomere organization | 0.0050 | MF |
| **73** | GO:0048015 | phosphatidylinositol-mediated signaling | 0.0055 | MF |
| **74** | GO:0051050 | positive regulation of transport | 0.0240 | MF |
| **75** | GO:0009581 | detection of external stimulus | 0.0020 | MF |
| **76** | GO:0009582 | detection of abiotic stimulus | 0.0020 | MF |
| **77** | GO:0010038 | response to metal ion | 0.0050 | MF |
| **78** | GO:0007005 | mitochondrion organization | 0.0088 | MF |
| **79** | GO:0050708 | regulation of protein secretion | 0.0394 | MF |
| **80** | GO:0002791 | regulation of peptide secretion | 0.0394 | MF |
| **81** | GO:0009895 | negative regulation of catabolic process | 0.0104 | MF |
| **82** | GO:0006914 | autophagy | 0.0109 | MF |
| **83** | GO:0006936 | muscle contraction | 0.00925 | MF(W) |
| **84** | GO:0050714 | positive regulation of protein secretion | 0.00087 | MF(W) |
| **85** | GO:0002793 | positive regulation of peptide secretion | 0.00087 | MF(W) |
| **86** | GO:0051592 | response to calcium ion | 0.00149 | MF(W) |
| **87** | GO:0010737 | protein kinase A signaling | 0.00149 | MF(W) |
| **88** | GO:0048769 | sarcomerogenesis | 0.00169 | MF(W) |
| **89** | GO:0035995 | detection of muscle stretch | 0.00169 | MF(W) |
| **90** | GO:0045214 | sarcomere organization | 0.00169 | MF(W) |
| **91** | GO:0055003 | cardiac myofibril assembly | 0.00169 | MF(W) |
| **92** | GO:0030240 | skeletal muscle thin filament assembly | 0.00169 | MF(W) |
| **93** | GO:0030241 | skeletal muscle myosin thick filament assembly | 0.00169 | MF(W) |
| **94** | GO:0060048 | cardiac muscle contraction | 0.00669 | MF(W) |
| **95** | GO:0001756 | somitogenesis | 0.00677 | MF(W) |
| **96** | GO:0021591 | ventricular system development | 0.00677 | MF(W) |
| **97** | GO:0055008 | cardiac muscle tissue morphogenesis | 0.00758 | MF(W) |
| **98** | GO:0003300 | cardiac muscle hypertrophy | 0.00764 | MF(W) |
| **99** | GO:0045859 | regulation of protein kinase activity | 0.00145 | MF(W) |
| **100** | GO:0001701 | in utero embryonic development | 0.01498 | MF(W) |

**Table S2.** Table of gene ontology biological process terms from downregulated genes from Inland Silverside larvae after 21-day exposure to the control and the ten treatmentS µTP, µTP(W), nTP, nTP(W), µPLA, µPLA(W), nPLA, nPLA(W) exposures contained 50 particles/mL and MF & MF(W) contained 30 particles/mL. TP = tire particle, PLA = polylactic acid, MF = polyester microfiber, W = weathered.

|  | **GO.ID** | **Term** | **classicKS** | **treatment** |
| --- | --- | --- | --- | --- |
| **1** | GO:0009968 | negative regulation of signal transduction | 0.0058 | µPLA |
| **2** | GO:0032268 | regulation of cellular protein metabolic process | 0.0062 | µPLA |
| **3** | GO:0008380 | RNA splicing | 0.0131 | µPLA |
| **4** | GO:0046496 | nicotinamide nucleotide metabolic process | 0.0201 | µPLA |
| **5** | GO:0009968 | negative regulation of signal transduction | 7E-04 | nPLA |
| **6** | GO:0071310 | cellular response to organic substance | 0.0102 | nPLA |
| **7** | GO:0045597 | positive regulation of cell differentiation | 0.0244 | nPLA |
| **8** | GO:0051707 | response to other organism | 0.0118 | nPLA |
| **9** | GO:0080135 | regulation of cellular response to stress | 0.0161 | nPLA |
| **10** | GO:0034097 | response to cytokine | 0.0247 | nPLA |
| **11** | GO:0002252 | immune effector process | 0.0251 | nPLA |
| **12** | GO:0031323 | regulation of cellular metabolic process | 0.0205 | nPLA(W) |
| **13** | GO:2000112 | regulation of cellular macromolecule biosynthetic process | 0.0095 | nPLA(W) |
| **14** | GO:0046496 | nicotinamide nucleotide metabolic process | 0.0124 | nPLA(W) |
| **15** | GO:0006955 | immune response | 0.0133 | nPLA(W) |
| **16** | GO:0042330 | taxis | 0.0187 | nPLA(W) |
| **17** | GO:0007017 | microtubule-based process | 0.0254 | nPLA(W) |
| **18** | GO:0031331 | positive regulation of cellular cataboli... | 0.0224 | nPLA(W) |
| **19** | GO:0042063 | gliogenesis | 0.0271 | nPLA(W) |
| **20** | GO:0042127 | regulation of cell population proliferation | 0.0021 | µPLA(W) |
| **21** | GO:0009968 | negative regulation of signal transduction | 0.0021 | µPLA(W) |
| **22** | GO:0006936 | muscle contraction | 0.0173 | µPLA(W) |
| **23** | GO:0045937 | positive regulation of phosphate metabolism | 0.0404 | µPLA(W) |
| **24** | GO:0009887 | animal organ morphogenesis | 0.0177 | µPLA(W) |
| **25** | GO:0006325 | chromatin organization | 0.0238 | µPLA(W) |
| **26** | GO:0044419 | biological process involved in interspecies interaction between organisms | 0.0197 | µPLA(W) |
| **27** | GO:0071310 | cellular response to organic substance | 0.0045 | µPLA(W) |
| **28** | GO:0080135 | regulation of cellular response to stress | 0.0055 | µTP |
| **29** | GO:0071407 | cellular response to organic cyclic compound | 0.0078 | µTP |
| **30** | GO:1903320 | regulation of protein modification by small protein conjugation or removal | 0.0162 | µTP |
| **31** | GO:0000398 | mRNA splicing, via spliceosome | 0.0212 | µTP |
| **32** | GO:0031589 | cell-substrate adhesion | 0.00382 | µTP(W) |
| **33** | GO:0097435 | supramolecular fiber organization | 0.00841 | µTP(W) |
| **34** | GO:0045859 | regulation of protein kinase activity | 0.01882 | µTP(W) |
| **35** | GO:0042692 | muscle cell differentiation | 0.00263 | µTP(W) |
| **36** | GO:0009968 | negative regulation of signal transduction | 0.00933 | µTP(W) |
| **37** | GO:0010467 | gene expression | 0.01936 | µTP(W) |
| **38** | GO:0034097 | response to cytokine | 0.03448 | µTP(W) |
| **39** | GO:0030155 | regulation of cell adhesion | 0.03575 | µTP(W) |
| **40** | GO:0071310 | cellular response to organic substance | 0.00066 | µTP(W) |
| **41** | GO:0098542 | defense response to other organism | 0.04485 | µTP(W) |
| **42** | GO:0034660 | ncRNA metabolic process | 0.0049 | nTP |
| **43** | GO:0043043 | peptide biosynthetic process | 0.0265 | nTP |
| **44** | GO:0006412 | translation | 0.0263 | nTP |
| **45** | GO:0140694 | non-membrane-bounded organelle assembly | 0.0144 | nTP |
| **46** | GO:0032268 | regulation of cellular protein metabolic process | 0.0181 | nTP |
| **47** | GO:0043066 | negative regulation of apoptotic process | 0.0224 | nTP |
| **48** | GO:0009968 | negative regulation of signal transduction | 0.011 | nTP |
| **49** | GO:0008284 | positive regulation of cell population proliferation | 0.0335 | nTP |
| **50** | GO:0001932 | regulation of protein phosphorylation | 0.0281 | nTP |
| **51** | GO:0043085 | positive regulation of catalytic activity | 0.0469 | nTP |
| **52** | GO:0007517 | muscle organ development | 0.00026 | nTP(W) |
| **53** | GO:0009628 | response to abiotic stimulus | 0.00052 | nTP(W) |
| **54** | GO:0032268 | regulation of cellular protein metabolic process | 0.00077 | nTP(W) |
| **55** | GO:0030239 | myofibril assembly | 0.00148 | nTP(W) |
| **56** | GO:0048729 | tissue morphogenesis | 0.00188 | nTP(W) |
| **57** | GO:0003007 | heart morphogenesis | 0.00269 | nTP(W) |
| **58** | GO:0006941 | striated muscle contraction | 0.01294 | nTP(W) |
| **59** | GO:0001701 | in utero embryonic development | 0.00457 | nTP(W) |
| **60** | GO:0016570 | histone modification | 0.00594 | nTP(W) |
| **61** | GO:0031399 | regulation of protein modification process | 0.04612 | nTP(W) |
| **62** | GO:0033554 | cellular response to stress | 0.0243 | nTP(W) |
| **63** | GO:0043009 | chordate embryonic development | 0.0149 | nTP(W) |
| **64** | GO:0016570 | histone modification | 0.012 | MF |
| **65** | GO:0048585 | negative regulation of response to stimulus | 0.019 | MF(W) |
| **66** | GO:0080134 | regulation of response to stress | 0.016 | MF(W) |
| **67** | GO:0010628 | positive regulation of gene expression | 0.012 | MF(W) |

**Table S3.** Table of gene ontology molecular function terms from upregulated genes from Inland Silverside larvae after 21-day exposure to the control and the ten treatmentS µTP, µTP(W), nTP, nTP(W), µPLA, µPLA(W), nPLA, nPLA(W) exposures contained 50 particles/mL and MF & MF(W) contained 30 particles/mL. TP = tire particle, PLA = polylactic acid, MF = polyester microfiber, W = weathered.

|  | **GO.ID** | **Term** | **classicKS** | **treatment** |
| --- | --- | --- | --- | --- |
| **1** | GO:0097493 | structural molecule activity conferring elasticity | 0.0026 | µPLA |
| **2** | GO:0005251 | delayed rectifier potassium channel activity | 0.0034 | µPLA |
| **3** | GO:0031177 | phosphopantetheine binding | 0.0044 | µPLA |
| **4** | GO:0008693 | 3-hydroxydecanoyl-[acyl-carrier-protein] dehydratase activity | 0.0044 | µPLA |
| **5** | GO:0047451 | 3-hydroxyoctanoyl-[acyl-carrier-protein] dehydratase activity | 0.0044 | µPLA |
| **6** | GO:0008659 | (3R)-hydroxymyristoyl-[acyl-carrier-protein] dehydratase activity | 0.0044 | µPLA |
| **7** | GO:0016295 | myristoyl-[acyl-carrier-protein] hhydrolase activity | 0.0044 | µPLA |
| **8** | GO:0016296 | palmitoyl-[acyl-carrier-protein] hydrolase activity | 0.0044 | µPLA |
| **9** | GO:0047117 | enoyl-[acyl-carrier-protein] reductase (NADPH, A-specific) activity | 0.0044 | µPLA |
| **10** | GO:0004320 | oleoyl-[acyl-carrier-protein] hydrolase activity | 0.0044 | µPLA |
| **11** | GO:0004313 | [acyl-carrier-protein] S-acetyltransferase activity | 0.0044 | µPLA |
| **12** | GO:0004314 | [acyl-carrier-protein] S-malonyltransferase activity | 0.0044 | µPLA |
| **13** | GO:0004315 | 3-oxoacyl-[acyl-carrier-protein] synthase activity | 0.0044 | µPLA |
| **14** | GO:0004316 | 3-oxoacyl-[acyl-carrier-protein] reductase (NADPH) activity | 0.0044 | µPLA |
| **15** | GO:0004317 | 3-hydroxypalmitoyl-[acyl-carrier-protein] dehydratase activity | 0.0044 | µPLA |
| **16** | GO:0008201 | heparin binding | 0.0061 | µPLA |
| **17** | GO:0002020 | protease binding | 0.0118 | µPLA |
| **18** | GO:0031433 | telethonin binding | 0.0118 | µPLA |
| **19** | GO:0051371 | muscle alpha-actinin binding | 0.0118 | µPLA |
| **20** | GO:0005546 | phosphatidylinositol-4,5-bisphosphate binding | 0.0159 | µPLA |
| **21** | GO:0044877 | protein-containing complex binding | 0.036 | nPLA |
| **22** | GO:0016887 | ATP hydrolysis activity | 0.010 | nPLA |
| **23** | GO:0015399 | primary active transmembrane transporter activity | 0.015 | nPLA |
| **24** | GO:0008307 | structural constituent of muscle | 0.035 | nPLA |
| **25** | GO:0019901 | protein kinase binding | 0.039 | nPLA |
| **26** | GO:0003713 | transcription coactivator activity | 0.041 | nPLA |
| **27** | GO:0051371 | muscle alpha-actinin binding | 0.00033 | nPLA(W) |
| **28** | GO:0097493 | structural molecule activity conferring elasticity | 0.00033 | nPLA(W) |
| **29** | GO:0031433 | telethonin binding | 0.00033 | nPLA(W) |
| **30** | GO:0002020 | protease binding | 0.00033 | nPLA(W) |
| **31** | GO:0005509 | calcium ion binding | 0.00089 | nPLA(W) |
| **32** | GO:0004713 | protein tyrosine kinase activity | 0.00099 | nPLA(W) |
| **33** | GO:0008307 | structural constituent of muscle | 0.00182 | nPLA(W) |
| **34** | GO:0030506 | ankyrin binding | 0.00237 | nPLA(W) |
| **35** | GO:0051015 | actin filament binding | 0.00275 | nPLA(W) |
| **36** | GO:0005516 | calmodulin binding | 0.00278 | nPLA(W) |
| **37** | GO:0043621 | protein self-association | 0.01566 | nPLA(W) |
| **38** | GO:0106310 | protein serine kinase activity | 0.01893 | nPLA(W) |
| **39** | GO:0019901 | protein kinase binding | 0.01912 | nPLA(W) |
| **40** | GO:0004674 | protein serine/threonine kinase activity | 0.02694 | nPLA(W) |
| **41** | GO:0042802 | identical protein binding | 0.03360 | nPLA(W) |
| **42** | GO:0043168 | anion binding | 0.02110 | nPLA(W) |
| **43** | GO:0036094 | small molecule binding | 0.00620 | nPLA(W) |
| **44** | GO:0005215 | transporter activity | 0.023 | µPLA(W) |
| **45** | GO:0022804 | active transmembrane transporter activit... | 0.035 | µTP |
| **46** | GO:0008092 | cytoskeletal protein binding | 0.035 | µTP |
| **47** | GO:1901681 | sulfur compound binding | 0.00034 | µTP(W) |
| **48** | GO:0005102 | signaling receptor binding | 0.00334 | µTP(W) |
| **49** | GO:0097367 | carbohydrate derivative binding | 0.00094 | µTP(W) |
| **50** | GO:0004713 | protein tyrosine kinase activity | 0.02909 | µTP(W) |
| **51** | GO:0002020 | protease binding | 0.0024 | nTP |
| **52** | GO:0043621 | protein self-association | 0.0026 | nTP |
| **53** | GO:0008307 | structural constituent of muscle | 0.0081 | nTP |
| **54** | GO:0005516 | calmodulin binding | 0.0117 | nTP |
| **55** | GO:0051371 | muscle alpha-actinin binding | 0.0117 | nTP |
| **56** | GO:0031433 | telethonin binding | 0.0117 | nTP |
| **57** | GO:0097493 | structural molecule activity conferring elasticity | 0.0117 | nTP |
| **58** | GO:0051015 | actin filament binding | 0.0171 | nTP |
| **59** | GO:0140096 | catalytic activity, acting on a protein | 0.0154 | nTP |
| **60** | GO:0046873 | metal ion transmembrane transporter activity | 0.04654 | nTP(W) |
| **61** | GO:0008307 | structural constituent of muscle | 0.00182 | nTP(W) |
| **62** | GO:0097493 | structural molecule activity conferring elasticity | 0.00033 | nTP(W) |
| **63** | GO:0008307 | structural constituent of muscle | 0.0056 | MF |
| **64** | GO:0044877 | protein-containing complex binding | 0.0486 | MF |
| **65** | GO:0043621 | protein self-association | 0.0092 | MF |
| **66** | GO:0002020 | protease binding | 0.0142 | MF |
| **67** | GO:0097367 | carbohydrate derivative binding | 0.0154 | MF |
| **68** | GO:0005509 | calcium ion binding | 0.0156 | MF |
| **69** | GO:0051371 | muscle alpha-actinin binding | 0.0217 | MF |
| **70** | GO:0097493 | structural molecule activity conferring elasticity | 0.0217 | MF |
| **71** | GO:0031433 | telethonin binding | 0.0217 | MF |
| **72** | GO:0051015 | actin filament binding | 0.0440 | MF |
| **73** | GO:0004713 | protein tyrosine kinase activity | 0.0485 | MF |
| **74** | GO:0008307 | structural constituent of muscle | 0.0016 | MF(W) |
| **75** | GO:0051015 | actin filament binding | 0.0021 | MF(W) |
| **76** | GO:0002020 | protease binding | 0.0034 | MF(W) |
| **77** | GO:0051371 | muscle alpha-actinin binding | 0.0036 | MF(W) |
| **78** | GO:0097493 | structural molecule activity conferring elasticity | 0.0036 | MF(W) |
| **79** | GO:0031433 | telethonin binding | 0.0036 | MF(W) |
| **80** | GO:0005509 | calcium ion binding | 0.0044 | MF(W) |
| **81** | GO:0004713 | protein tyrosine kinase activity | 0.0045 | MF(W) |
| **82** | GO:0043621 | protein self-association | 0.0110 | MF(W) |
| **83** | GO:0005200 | structural constituent of cytoskeleton | 0.0244 | MF(W) |
| **84** | GO:0005516 | calmodulin binding | 0.0350 | MF(W) |
| **85** | GO:0042802 | identical protein binding | 0.0106 | MF(W) |
| **86** | GO:0046873 | metal ion transmembrane transporter activity | 0.0021 | MF(W) |


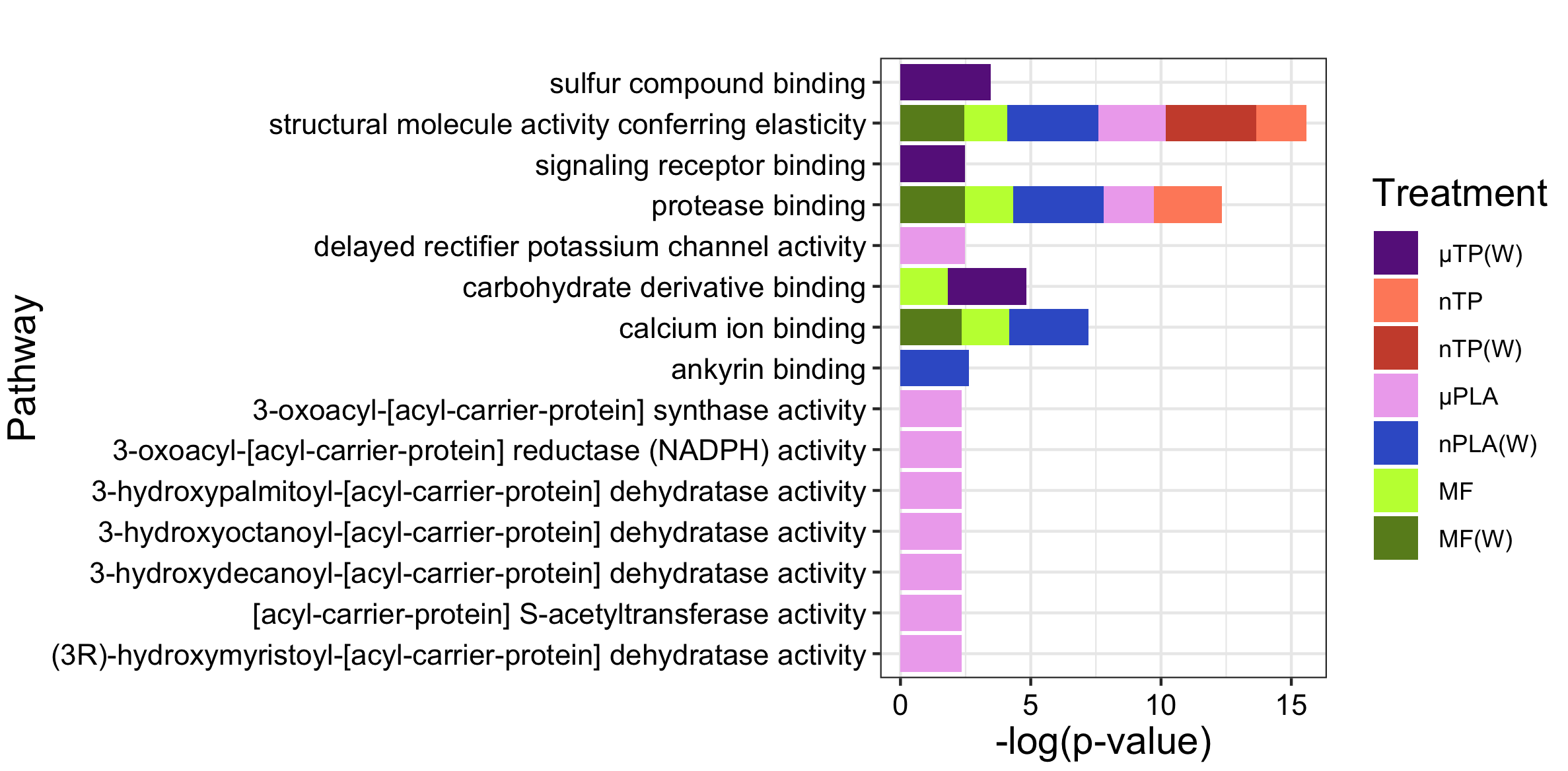


**Figure S8.** Stacked bar plot showing the top 15 upregulated GO molecular function process terms in Inland Silverside larvae following 21-day exposure to all the MNP treatments. Not all treatments are displayed as they were not represented by the top 15 GO terms. TP = tire particle (exposed at 50 p/mL); PLA = polylactic acid (exposed at 50 p/mL); MF = polyester microfiber (exposed at 30 p/mL). W = weathered; particles were weathered under UV A, B, and C light on a shaker at 15 PSU to simulate wave action. p < 0.05, logFC > 0.

**Table S4**. Table of gene ontology molecular function terms from downregulated genes from Inland Silverside larvae after 21-day exposure to the control and the ten treatmentS µTP, µTP(W), nTP, nTP(W), µPLA, µPLA(W), nPLA, nPLA(W) exposures contained 50 particles/mL and MF & MF(W) contained 30 particles/mL. TP = tire particle, PLA = polylactic acid, MF = polyester microfiber, W = weathered.

|  | **GO.ID** | **Term** | **classicKS** | **treatment** |
| --- | --- | --- | --- | --- |
| **1** | GO:0003774 | cytoskeletal motor activity | 0.03 | upla |
| **2** | GO:0000981 | DNA-binding transcription factor activity | 0.048 | upla |
| **3** | GO:0043021 | ribonucleoprotein complex binding | 0.017 | npla_w |
| **4** | GO:0017111 | ribonucleoside triphosphate phosphatase activity | 0.035 | npla_w |
| **5** | GO:0003677 | DNA binding | 0.022 | npla_w |
| **6** | GO:0005215 | transporter activity | 0.023 | upla_w |
| **7** | GO:0003700 | DNA-binding transcription factor activity | 0.0092 | utp |
| **8** | GO:0008134 | transcription factor binding | 0.0186 | utp |
| **9** | GO:0051015 | actin filament binding | 0.0093 | utp_w |
| **10** | GO:0016740 | transferase activity | 0.0362 | utp_w |
| **11** | GO:0003700 | DNA-binding transcription factor activity | 0.0036 | utp_w |
| **12** | GO:0003676 | nucleic acid binding | 0.0311 | utp_w |
| **13** | GO:0003723 | RNA binding | 0.0051 | ntp |
| **14** | GO:0032555 | purine ribonucleotide binding | 0.0415 | ntp |
| **15** | GO:0035639 | purine ribonucleoside triphosphate binding | 0.043 | ntp |
| **16** | GO:0017111 | ribonucleoside triphosphate phosphatase activity | 0.0218 | ntp |
| **17** | GO:0051015 | actin filament binding | 0.0072 | ntp_w |
| **18** | GO:0005509 | calcium ion binding | 0.0426 | ntp_w |
| **19** | GO:0003676 | nucleic acid binding | 0.039 | mf |
| **20** | GO:0005509 | calcium ion binding | 0.0044 | mf_w |
| **21** | GO:0051015 | actin filament binding | 0.0021 | mf_w |


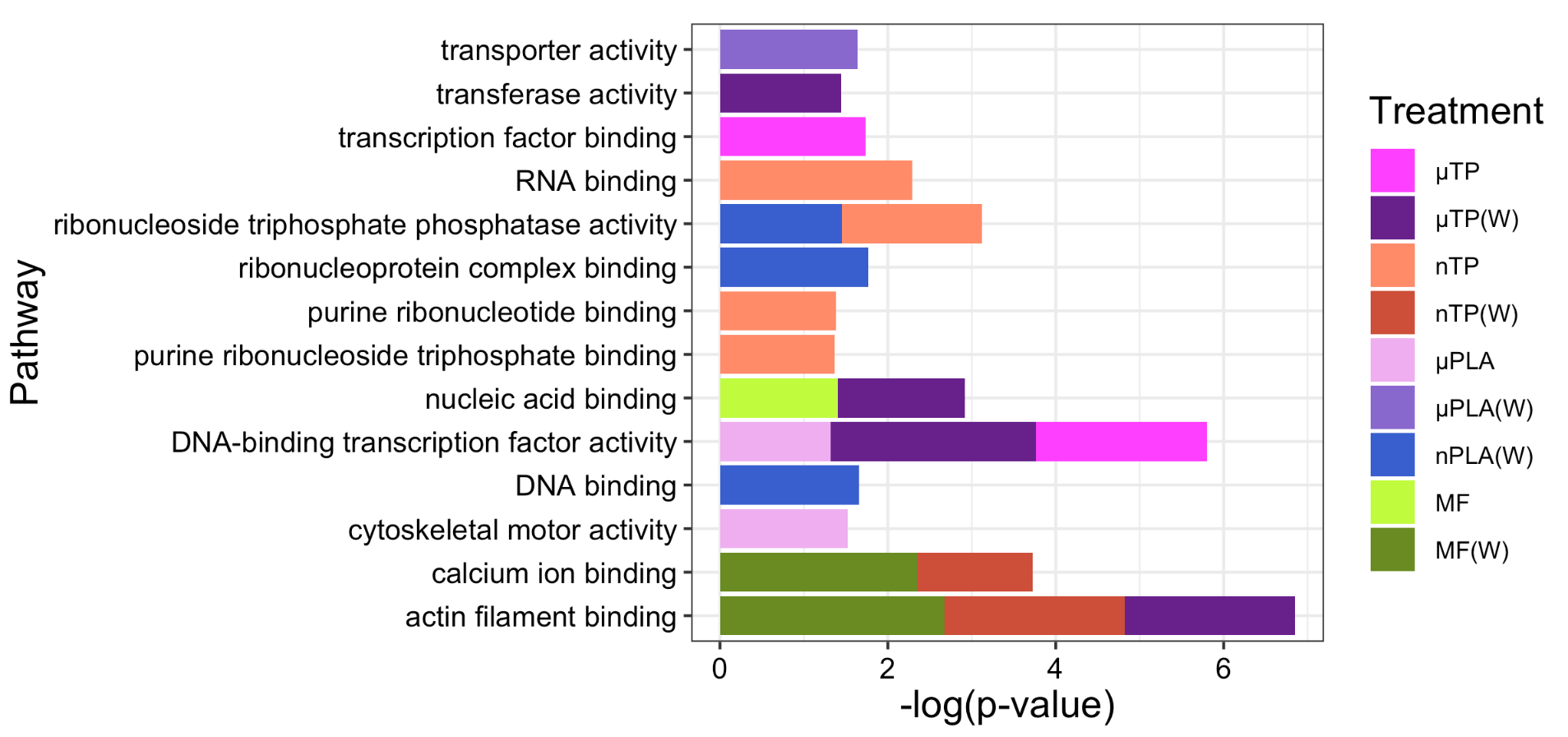


**Figure S9.** Stacked bar plot showing the top 15 downregulated GO molecular function process terms in Inland Silverside larvae following 21-day exposure to all the MNP treatmentS Not all treatments are displayed as they were not represented by the top 15 GO termS TP = tire particle (exposed at 50 p/mL); PLA = polylactic acid (exposed at 50 p/mL); MF = polyester microfiber (exposed at 30 p/mL). W = weathered; particles were weathered under UV A, B, and C light on a shaker at 15 PSU to simulate wave action. p < 0.05, logFC < 0.

**Table S5.** Table of gene ontology cellular component terms from upregulated genes from Inland Silverside larvae after 21-day exposure to the control and the ten treatmentS µTP, µTP(W), nTP, nTP(W), µPLA, µPLA(W), nPLA, nPLA(W) exposures contained 50 particles/mL and MF & MF(W) contained 30 particles/mL. TP = tire particle, PLA = polylactic acid, MF = polyester microfiber, W = weathered.

|  | **GO.ID** | **Term** | **classicKS** | **treatment** |
| --- | --- | --- | --- | --- |
| **1** | GO:0045296 | cadherin binding | 1.7E-08 | µPLA |
| **2** | GO:0060589 | nucleoside-triphosphatase regulator activity | 1.2E-05 | µPLA |
| **3** | GO:0060590 | ATPase regulator activity | 1.3E-05 | µPLA |
| **4** | GO:0051117 | ATPase binding | 3.1E-05 | µPLA |
| **5** | GO:0008656 | cysteine-type endopeptidase activator activity involved in apoptotic process | 8.6E-05 | µPLA |
| **6** | GO:0140223 | general transcription initiation factor activity | 0.00014 | µPLA |
| **7** | GO:0005518 | collagen binding | 0.00016 | µPLA |
| **8** | GO:0003723 | RNA binding | 0.00048 | µPLA |
| **9** | GO:0003712 | transcription coregulator activity | 0.02129 | µPLA |
| **10** | GO:0003925 | G protein activity | 0.00053 | µPLA |
| **11** | GO:0044877 | protein-containing complex binding | 8.5E-07 | µPLA |
| **12** | GO:0030527 | structural constituent of chromatin | 0.00072 | µPLA |
| **13** | GO:0008428 | ribonuclease inhibitor activity | 0.00076 | µPLA |
| **14** | GO:0046875 | ephrin receptor binding | 0.00094 | µPLA |
| **15** | GO:0019003 | GDP binding | 0.00143 | µPLA |
| **16** | GO:0005471 | ATP:ADP antiporter activity | 0.00146 | µPLA |
| **17** | GO:0030674 | protein-macromolecule adaptor activity | 0.04441 | µPLA |
| **18** | GO:0052650 | NADP-retinol dehydrogenase activity | 0.00182 | µPLA |
| **19** | GO:0017124 | SH3 domain binding | 0.00205 | µPLA |
| **20** | GO:0005126 | cytokine receptor binding | 0.00393 | µPLA |
| **21** | GO:0045296 | cadherin binding | 8E-08 | nPLA |
| **22** | GO:0003723 | RNA binding | 9.5E-05 | nPLA |
| **23** | GO:0001046 | core promoter sequence-specific DNA binding | 9.2E-05 | nPLA |
| **24** | GO:0003690 | double-stranded DNA binding | 0.00012 | nPLA |
| **25** | GO:0140223 | general transcription initiation factor activity | 0.00015 | nPLA |
| **26** | GO:0003712 | transcription coregulator activity | 0.00059 | nPLA |
| **27** | GO:0003713 | transcription coactivator activity | 0.00047 | nPLA |
| **28** | GO:0001221 | transcription coregulator binding | 0.00379 | nPLA |
| **29** | GO:0030674 | protein-macromolecule adaptor activity | 0.01156 | nPLA |
| **30** | GO:0060590 | ATPase regulator activity | 0.00039 | nPLA |
| **31** | GO:0051117 | ATPase binding | 0.00041 | nPLA |
| **32** | GO:0140297 | DNA-binding transcription factor binding | 0.00071 | nPLA |
| **33** | GO:0008656 | cysteine-type endopeptidase activator activity involved in apoptotic process | 0.00086 | nPLA |
| **34** | GO:0008022 | protein C-terminus binding | 0.00107 | nPLA |
| **35** | GO:0052650 | NADP-retinol dehydrogenase activity | 0.00109 | nPLA |
| **36** | GO:0061629 | RNA polymerase II-specific DNA-binding transcription factor binding | 0.03640 | nPLA |
| **37** | GO:0046875 | ephrin receptor binding | 0.00131 | nPLA |
| **38** | GO:0140101 | catalytic activity, acting on a tRNA | 0.05138 | nPLA |
| **39** | GO:0060589 | nucleoside-triphosphatase regulator activity | 0.00037 | nPLA |
| **40** | GO:0005471 | ATP:ADP antiporter activity | 0.00269 | nPLA |
| **41** | GO:0045296 | cadherin binding | 8E-08 | nPLA(W) |
| **42** | GO:0001221 | transcription coregulator binding | 0.00316 | nPLA(W) |
| **43** | GO:0003723 | RNA binding | 0.00269 | nPLA(W) |
| **44** | GO:0051117 | ATPase binding | 6.7E-05 | nPLA(W) |
| **45** | GO:0008656 | cysteine-type endopeptidase activator activity involved in apoptotic process | 7.1E-05 | nPLA(W) |
| **46** | GO:0060590 | ATPase regulator activity | 8.7E-05 | nPLA(W) |
| **47** | GO:0003925 | G protein activity | 0.00012 | nPLA(W) |
| **48** | GO:0001046 | core promoter sequence-specific DNA binding | 0.00024 | nPLA(W) |
| **49** | GO:0043539 | protein serine/threonine kinase activator activity | 0.00073 | nPLA(W) |
| **50** | GO:0003684 | damaged DNA binding | 0.00087 | nPLA(W) |
| **51** | GO:0060589 | nucleoside-triphosphatase regulator activity | 8.2E-05 | nPLA(W) |
| **52** | GO:0008428 | ribonuclease inhibitor activity | 0.00140 | nPLA(W) |
| **53** | GO:0051059 | NF-kappaB binding | 0.00144 | nPLA(W) |
| **54** | GO:0016922 | nuclear receptor binding | 0.19153 | nPLA(W) |
| **55** | GO:0051427 | hormone receptor binding | 0.00177 | nPLA(W) |
| **56** | GO:0031490 | chromatin DNA binding | 0.00193 | nPLA(W) |
| **57** | GO:0140677 | molecular function activator activity | 0.00197 | nPLA(W) |
| **58** | GO:0016251 | RNA polymerase II general transcription initiation factor activity | 0.00198 | nPLA(W) |
| **59** | GO:0000981 | DNA-binding transcription factor activity, RNA polymerase II-specific | 0.01293 | nPLA(W) |
| **60** | GO:0005471 | ATP:ADP antiporter activity | 0.00261 | nPLA(W) |
| **61** | GO:0045296 | cadherin binding | 5.9E-08 | µPLA(W) |
| **62** | GO:0060589 | nucleoside-triphosphatase regulator activity | 1.6E-05 | µPLA(W) |
| **63** | GO:0060590 | ATPase regulator activity | 1.7E-05 | µPLA(W) |
| **64** | GO:0051117 | ATPase binding | 2.8E-05 | µPLA(W) |
| **65** | GO:0003723 | RNA binding | 0.00042 | µPLA(W) |
| **66** | GO:0003925 | G protein activity | 0.00026 | µPLA(W) |
| **67** | GO:0001228 | DNA-binding transcription activator activity, RNA polymerase II-specific | 0.00028 | µPLA(W) |
| **68** | GO:0046875 | ephrin receptor binding | 0.00084 | µPLA(W) |
| **69** | GO:0030674 | protein-macromolecule adaptor activity | 0.03980 | µPLA(W) |
| **70** | GO:0008022 | protein C-terminus binding | 0.00114 | µPLA(W) |
| **71** | GO:0001221 | transcription coregulator binding | 0.01902 | µPLA(W) |
| **72** | GO:0031625 | ubiquitin protein ligase binding | 0.00143 | µPLA(W) |
| **73** | GO:0051879 | Hsp90 protein binding | 0.00144 | µPLA(W) |
| **74** | GO:0001046 | core promoter sequence-specific DNA binding | 0.00176 | µPLA(W) |
| **75** | GO:0051427 | hormone receptor binding | 0.00178 | µPLA(W) |
| **76** | GO:0005518 | collagen binding | 0.00181 | µPLA(W) |
| **77** | GO:0016251 | RNA polymerase II general transcription initiation factor activity | 0.00200 | µPLA(W) |
| **78** | GO:0001099 | basal RNA polymerase II transcription machinery binding | 0.04466 | µPLA(W) |
| **79** | GO:0140297 | DNA-binding transcription factor binding | 0.00852 | µPLA(W) |
| **80** | GO:0016922 | nuclear receptor binding | 0.20759 | µPLA(W) |
| **81** | GO:0045296 | cadherin binding | 3.3E-07 | µTP |
| **82** | GO:0008047 | enzyme activator activity | 0.00749 | µTP |
| **83** | GO:0003723 | RNA binding | 0.00209 | µTP |
| **84** | GO:0051117 | ATPase binding | 7.5E-05 | µTP |
| **85** | GO:0031490 | chromatin DNA binding | 0.00011 | µTP |
| **86** | GO:0001221 | transcription coregulator binding | 0.01371 | µTP |
| **87** | GO:0003682 | chromatin binding | 0.00012 | µTP |
| **88** | GO:0001228 | DNA-binding transcription activator activity, RNA polymerase II-specific | 0.00019 | µTP |
| **89** | GO:0001046 | core promoter sequence-specific DNA binding | 0.00021 | µTP |
| **90** | GO:0140223 | general transcription initiation factor activity | 0.00026 | µTP |
| **91** | GO:0060590 | ATPase regulator activity | 0.00052 | µTP |
| **92** | GO:0003690 | double-stranded DNA binding | 0.00200 | µTP |
| **93** | GO:0003712 | transcription coregulator activity | 0.00259 | µTP |
| **94** | GO:0003925 | G protein activity | 0.00102 | µTP |
| **95** | GO:0060589 | nucleoside-triphosphatase regulator activity | 0.00050 | µTP |
| **96** | GO:0140297 | DNA-binding transcription factor binding | 0.00264 | µTP |
| **97** | GO:0047485 | protein N-terminus binding | 0.00114 | µTP |
| **98** | GO:0030674 | protein-macromolecule adaptor activity | 0.12201 | µTP |
| **99** | GO:0046875 | ephrin receptor binding | 0.00124 | µTP |
| **100** | GO:0052650 | NADP-retinol dehydrogenase activity | 0.00132 | µTP |
| **101** | GO:0045296 | cadherin binding | 7.9E-10 | µTP(W) |
| **102** | GO:0008656 | cysteine-type endopeptidase activator activity involved in apoptotic process | 5.8E-05 | µTP(W) |
| **103** | GO:0030674 | protein-macromolecule adaptor activity | 0.00155 | µTP(W) |
| **104** | GO:0044877 | protein-containing complex binding | 1.2E-07 | µTP(W) |
| **105** | GO:0003682 | chromatin binding | 4.1E-05 | µTP(W) |
| **106** | GO:0140223 | general transcription initiation factor activity | 0.00011 | µTP(W) |
| **107** | GO:0051117 | ATPase binding | 0.00012 | µTP(W) |
| **108** | GO:0003723 | RNA binding | 0.00035 | µTP(W) |
| **109** | GO:0001221 | transcription coregulator binding | 0.00179 | µTP(W) |
| **110** | GO:0051427 | hormone receptor binding | 0.00074 | µTP(W) |
| **111** | GO:0003925 | G protein activity | 0.00078 | µTP(W) |
| **112** | GO:0046875 | ephrin receptor binding | 0.00084 | µTP(W) |
| **113** | GO:0008047 | enzyme activator activity | 0.00517 | µTP(W) |
| **114** | GO:0003690 | double-stranded DNA binding | 0.00190 | µTP(W) |
| **115** | GO:0008428 | ribonuclease inhibitor activity | 0.00105 | µTP(W) |
| **116** | GO:0016922 | nuclear receptor binding | 0.07188 | µTP(W) |
| **117** | GO:0001228 | DNA-binding transcription activator activity, RNA polymerase II-specific | 0.00129 | µTP(W) |
| **118** | GO:0060589 | nucleoside-triphosphatase regulator activity | 0.00136 | µTP(W) |
| **119** | GO:0060590 | ATPase regulator activity | 0.00142 | µTP(W) |
| **120** | GO:0043021 | ribonucleoprotein complex binding | 0.00967 | µTP(W) |
| **121** | GO:0045296 | cadherin binding | 1.7E-08 | nTP |
| **122** | GO:0003723 | RNA binding | 0.00059 | nTP |
| **123** | GO:0044877 | protein-containing complex binding | 6.4E-08 | nTP |
| **124** | GO:0003690 | double-stranded DNA binding | 0.00043 | nTP |
| **125** | GO:0008656 | cysteine-type endopeptidase activator activity involved in apoptotic process | 0.00015 | nTP |
| **126** | GO:0140223 | general transcription initiation factor activity | 0.00027 | nTP |
| **127** | GO:0060589 | nucleoside-triphosphatase regulator activity | 0.00031 | nTP |
| **128** | GO:0060590 | ATPase regulator activity | 0.00033 | nTP |
| **129** | GO:0001046 | core promoter sequence-specific DNA binding | 0.00051 | nTP |
| **130** | GO:0003712 | transcription coregulator activity | 0.02221 | nTP |
| **131** | GO:0051117 | ATPase binding | 0.00054 | nTP |
| **132** | GO:0052650 | NADP-retinol dehydrogenase activity | 0.00060 | nTP |
| **133** | GO:0047485 | protein N-terminus binding | 0.00103 | nTP |
| **134** | GO:0030674 | protein-macromolecule adaptor activity | 0.00600 | nTP |
| **135** | GO:0001228 | DNA-binding transcription activator activity, RNA polymerase II-specific | 0.00118 | nTP |
| **136** | GO:0003682 | chromatin binding | 0.00083 | nTP |
| **137** | GO:0046982 | protein heterodimerization activity | 0.00147 | nTP |
| **138** | GO:0070063 | RNA polymerase binding | 0.07191 | nTP |
| **139** | GO:0019003 | GDP binding | 0.00181 | nTP |
| **140** | GO:0015631 | tubulin binding | 0.01998 | nTP |
| **141** | GO:0045296 | cadherin binding | 8E-08 | nTP(W) |
| **142** | GO:0140223 | general transcription initiation factor activity | 0.27132 | nTP(W) |
| **143** | GO:0030527 | structural constituent of chromatin | 0.00697 | nTP(W) |
| **144** | GO:0008656 | cysteine-type endopeptidase activator activity involved in apoptotic process | 7.1E-05 | nTP(W) |
| **145** | GO:0051117 | ATPase binding | 6.7E-05 | nTP(W) |
| **146** | GO:0003723 | RNA binding | 0.00269 | nTP(W) |
| **147** | GO:0003712 | transcription coregulator activity | 0.00784 | nTP(W) |
| **148** | GO:0003690 | double-stranded DNA binding | 0.00142 | nTP(W) |
| **149** | GO:0031490 | chromatin DNA binding | 0.00193 | nTP(W) |
| **150** | GO:0060590 | ATPase regulator activity | 8.7E-05 | nTP(W) |
| **151** | GO:0005518 | collagen binding | 0.03845 | nTP(W) |
| **152** | GO:0001228 | DNA-binding transcription activator activity, RNA polymerase II-specific | 0.03378 | nTP(W) |
| **153** | GO:0030674 | protein-macromolecule adaptor activity | 0.03216 | nTP(W) |
| **154** | GO:0003925 | G protein activity | 0.00012 | nTP(W) |
| **155** | GO:0005471 | ATP:ADP antiporter activity | 0.00261 | nTP(W) |
| **156** | GO:0004725 | protein tyrosine phosphatase activity | 0.10131 | nTP(W) |
| **157** | GO:0001046 | core promoter sequence-specific DNA binding | 0.00024 | nTP(W) |
| **158** | GO:0008138 | protein tyrosine/serine/threonine phosphatase activity | 0.00445 | nTP(W) |
| **159** | GO:0046983 | protein dimerization activity | 0.35167 | nTP(W) |
| **160** | GO:0043565 | sequence-specific DNA binding | 0.36248 | nTP(W) |
| **161** | GO:0045296 | cadherin binding | 4.5E-08 | MF |
| **162** | GO:0060590 | ATPase regulator activity | 3.8E-05 | MF |
| **163** | GO:0140223 | general transcription initiation factor activity | 0.00013 | MF |
| **164** | GO:0003690 | double-stranded DNA binding | 0.00014 | MF |
| **165** | GO:0001046 | core promoter sequence-specific DNA binding | 0.00017 | MF |
| **166** | GO:0003723 | RNA binding | 0.00137 | MF |
| **167** | GO:0003925 | G protein activity | 0.00032 | MF |
| **168** | GO:0051117 | ATPase binding | 0.00033 | MF |
| **169** | GO:0008656 | cysteine-type endopeptidase activator activity involved in apoptotic process | 0.00041 | MF |
| **170** | GO:0001221 | transcription coregulator binding | 0.00951 | MF |
| **171** | GO:0008047 | enzyme activator activity | 0.00523 | MF |
| **172** | GO:0016922 | nuclear receptor binding | 0.00540 | MF |
| **173** | GO:0047485 | protein N-terminus binding | 0.00073 | MF |
| **174** | GO:0030527 | structural constituent of chromatin | 0.00073 | MF |
| **175** | GO:0001228 | DNA-binding transcription activator activity, RNA polymerase II-specific | 0.00096 | MF |
| **176** | GO:0044389 | ubiquitin-like protein ligase binding | 0.00097 | MF |
| **177** | GO:0003682 | chromatin binding | 0.00060 | MF |
| **178** | GO:0060589 | nucleoside-triphosphatase regulator activity | 3.6E-05 | MF |
| **179** | GO:0005471 | ATP:ADP antiporter activity | 0.00160 | MF |
| **180** | GO:0003713 | transcription coactivator activity | 0.01061 | MF |
| **181** | GO:0045296 | cadherin binding | 2.6E-08 | MF(W) |
| **182** | GO:0017124 | SH3 domain binding | 5.8E-05 | MF(W) |
| **183** | GO:0003723 | RNA binding | 0.00127 | MF(W) |
| **184** | GO:0140223 | general transcription initiation factor activity | 0.00012 | MF(W) |
| **185** | GO:0060589 | nucleoside-triphosphatase regulator activity | 0.00023 | MF(W) |
| **186** | GO:0060590 | ATPase regulator activity | 0.00025 | MF(W) |
| **187** | GO:0008656 | cysteine-type endopeptidase activator activity involved in apoptotic process | 0.00037 | MF(W) |
| **188** | GO:0001046 | core promoter sequence-specific DNA binding | 0.00042 | MF(W) |
| **189** | GO:0046875 | ephrin receptor binding | 0.00044 | MF(W) |
| **190** | GO:0019904 | protein domain specific binding | 0.00230 | MF(W) |
| **191** | GO:0001099 | basal RNA polymerase II transcription machinery binding | 0.08961 | MF(W) |
| **192** | GO:0030527 | structural constituent of chromatin | 0.00081 | MF(W) |
| **193** | GO:0003712 | transcription coregulator activity | 0.01686 | MF(W) |
| **194** | GO:0003690 | double-stranded DNA binding | 0.00285 | MF(W) |
| **195** | GO:0001228 | DNA-binding transcription activator activity, RNA polymerase II-specific | 0.00102 | MF(W) |
| **196** | GO:0001221 | transcription coregulator binding | 0.03082 | MF(W) |
| **197** | GO:0051117 | ATPase binding | 0.00126 | MF(W) |
| **198** | GO:0008022 | protein C-terminus binding | 0.00147 | MF(W) |
| **199** | GO:0030674 | protein-macromolecule adaptor activity | 0.03149 | MF(W) |
| **200** | GO:0008236 | serine-type peptidase activity | 0.04879 | MF(W) |


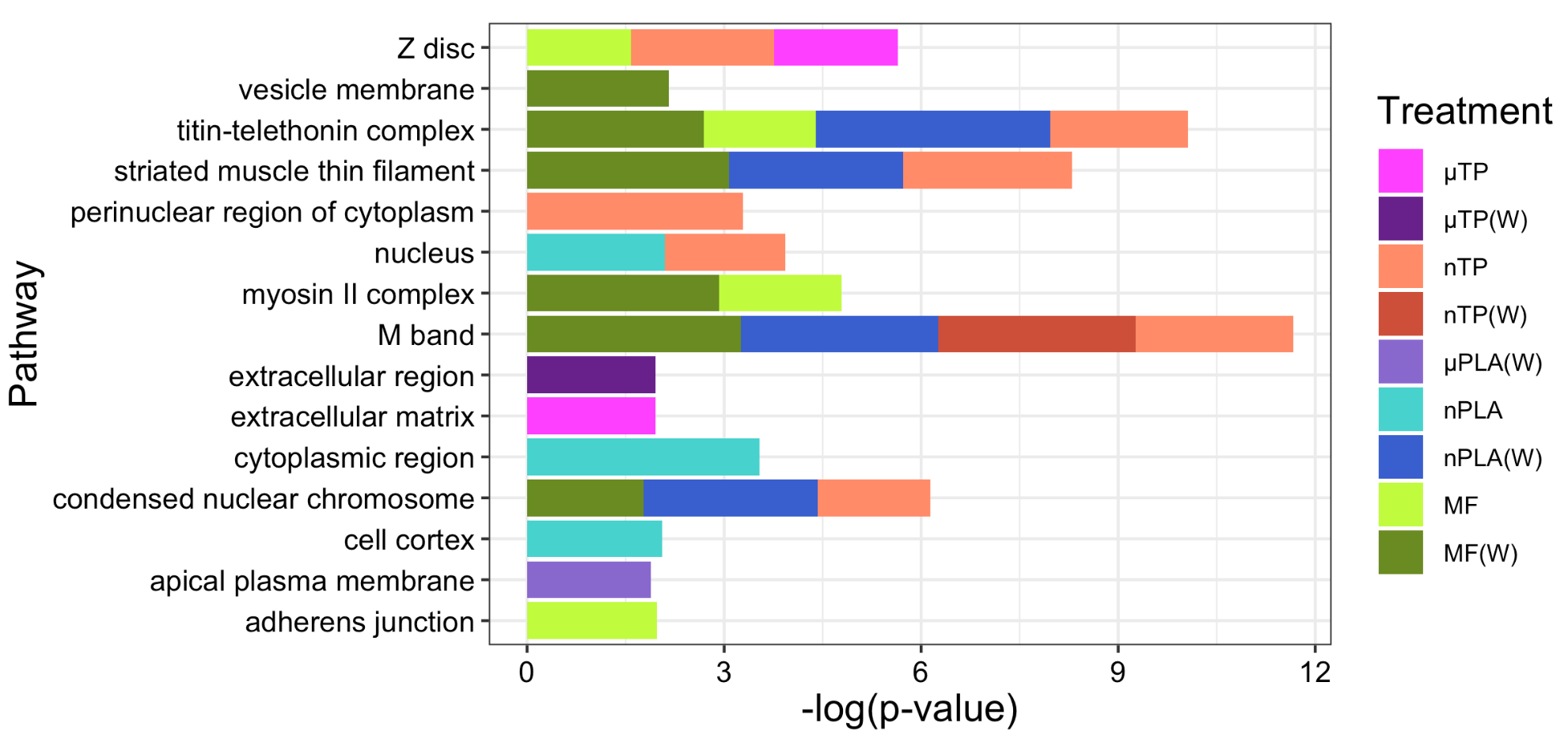


**Figure S10.** Stacked bar plot showing the top 15 upregulated GO cellular component terms in Inland Silverside larvae following 21-day exposure to all the MNP treatmentS Not all treatments are displayed as they were not represented by the top 15 GO termS TP = tire particle (exposed at 50 p/mL); PLA = polylactic acid (exposed at 50 p/mL); MF = polyester microfiber (exposed at 30 p/mL). W = weathered; particles were weathered under UV A, B, and C light on a shaker at 15 PSU to simulate wave action. p < 0.05, logFC < 0.

**Table S6.** Table of gene ontology cellular component terms from downregulated genes from Inland Silverside larvae after 21-day exposure to the control and the ten treatmentS µTP, µTP(W), nTP, nTP(W), µPLA, µPLA(W), nPLA, nPLA(W) exposures contained 50 particles/mL and MF & MF(W) contained 30 particles/mL. TP = tire particle, PLA = polylactic acid, MF = polyester microfiber, W = weathered.

|  | **GO.ID** | **Term** | **classicKS** | **treatment** |
| --- | --- | --- | --- | --- |
| **1** | GO:1990904 | ribonucleoprotein complex | 0.037 | µPLA |
| **2** | GO:0030016 | myofibril | 0.033 | µPLA |
| **3** | GO:0016607 | nuclear speck | 0.042 | nPLA |
| **4** | GO:0099512 | supramolecular fiber | 0.038 | nPLA(W) |
| **5** | GO:0016607 | nuclear speck | 0.049 | nPLA(W) |
| **6** | GO:0016607 | nuclear speck | 0.0071 | µTP |
| **7** | GO:0005815 | microtubule organizing center | 0.0129 | µTP |
| **8** | GO:0015629 | actin cytoskeleton | 0.0112 | µTP(W) |
| **9** | GO:0070062 | extracellular exosome | 0.0076 | µTP(W) |
| **10** | GO:0031012 | extracellular matrix | 0.0322 | µTP(W) |
| **11** | GO:1990904 | ribonucleoprotein complex | 0.0475 | µTP(W) |
| **12** | GO:0005783 | endoplasmic reticulum | 0.0225 | µTP(W) |
| **13** | GO:0005840 | ribosome | 0.013 | nTP |
| **14** | GO:0016607 | nuclear speck | 0.015 | nTP |
| **15** | GO:0005815 | microtubule organizing center | 0.017 | nTP |
| **16** | GO:1990904 | ribonucleoprotein complex | 0.038 | nTP |
| **17** | GO:0045202 | synapse | 0.011 | nTP |
| **18** | GO:0070013 | intracellular organelle lumen | 0.03 | nTP |
| **19** | GO:0030018 | Z disc | 0.003 | nTP(W) |
| **20** | GO:0070013 | intracellular organelle lumen | 0.0205 | nTP(W) |
| **21** | GO:0015629 | actin cytoskeleton | 0.0143 | nTP(W) |
| **22** | GO:0031981 | nuclear lumen | 0.0036 | nTP(W) |
| **23** | GO:0005815 | microtubule organizing center | 0.0096 | MF |
| **24** | GO:0045202 | synapse | 0.0464 | MF |
| **25** | GO:0099512 | supramolecular fiber | 0.004 | MF(W) |


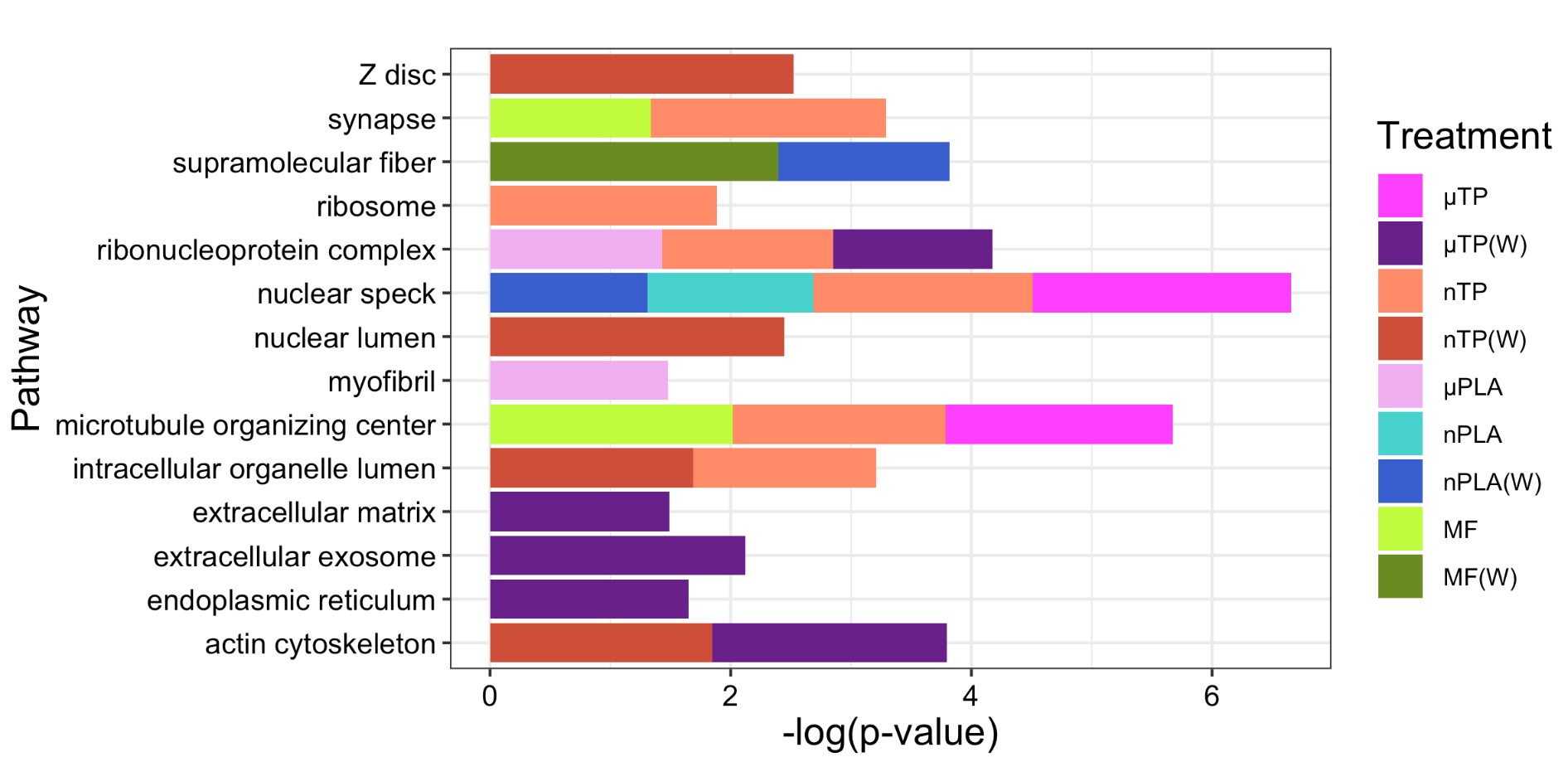


**Figure S11.** Stacked bar plot showing the top 15 downregulated GO cellular component terms in Inland Silverside larvae following 21-day exposure to all the MNP treatmentS Not all treatments are displayed as they were not represented by the top 15 GO termS TP = tire particle (exposed at 50 p/mL); PLA = polylactic acid (exposed at 50 p/mL); MF = polyester microfiber (exposed at 30 p/mL). W = weathered; particles were weathered under UV A, B, and C light on a shaker at 15 PSU to simulate wave action. p < 0.05, logFC < 0.
